# BCG vaccination elicits protection against *M*. *tuberculosis* infection mediated by two phases of T cell immunity

**DOI:** 10.1172/jci.insight.201000

**Published:** 2026-07-02

**Authors:** Abiola F. Ogunsola, Rocky Lai, Kelly Cavallo, Anthony V. Tran, Gillian L. Beamer, Samuel M. Behar

**Affiliations:** 1 Morningside Graduate School of Biomedical Sciences,; 2 Medical Scientist Training Program, and; 3 Department of Microbiology, University of Massachusetts Chan Medical School, Worcester, Massachusetts, USA.; 4 Texas Biomedical Research Institute, San Antonio, Texas, USA.

**Keywords:** Immunology, Infectious disease, Inflammation, T cells, Tuberculosis, Vaccines

## Abstract

Vaccine development for tuberculosis (TB) is a global priority. Our studies using Collaborative Cross (CC) mice show that genetic diversity influences the efficacy of BCG, the most widely used TB vaccine. BCG vaccination of CC042 mice reduced their lung bacillary burden and increased their survival following low-dose aerosol *Mycobacterium tuberculosis* infection (MTBI), despite impaired T cell trafficking due to a defective *Itgal* gene. BCG vaccination conferred early bacillary control that appeared to be independent of B cell or T cell recall responses following MTBI. In contrast, long-term survival of BCG-vaccinated CC042 mice after MTBI required T cells. Thus, CC042 mice reveal two phases of immunity induced by BCG: an early phase mediated by innate immunity or innate-like T cells and a later phase mediated by conventional memory CD4^+^ and/or CD8^+^ T cells. Although measurement of vaccine-induced protection 30 days after MTBI is a standard measure of vaccine efficacy in the TB model, this time point might be independent of memory T cells in CC042 mice. Our results suggest that vaccine-elicited innate/innate-like responses could have a larger role in protection than previously considered. The concordance between lung CFU, pathology, and survival makes CC042 mice useful for mechanistic studies on vaccine-induced immunity.

## Abstract 2

Using genetically diverse mice, we found that BCG vaccination mediates early protection against tuberculosis by nontraditional immune mechanisms; however, durable protection requires memory T cells.

## Graphical abstract

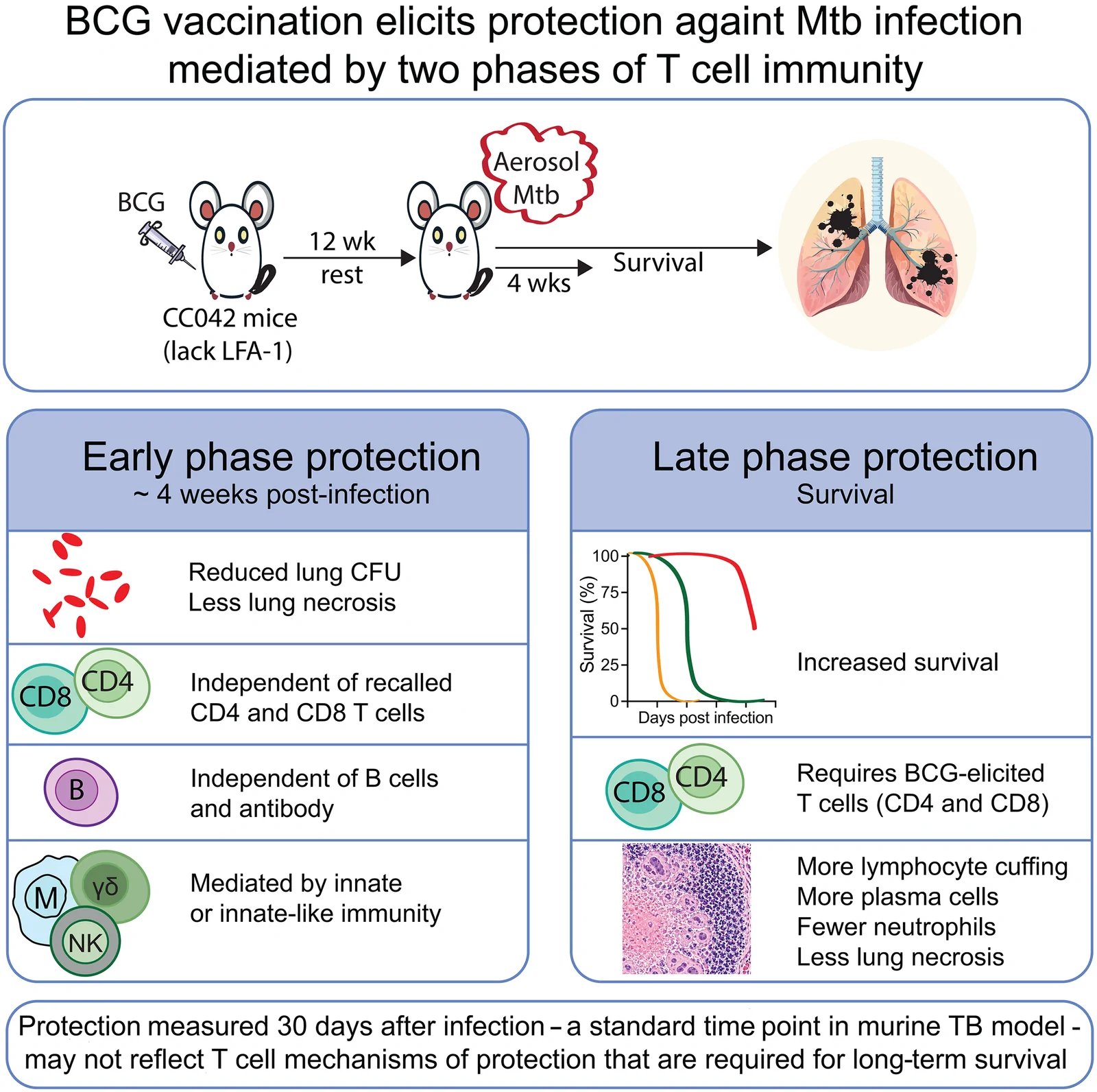

## Introduction

Tuberculosis (TB) is a chronic bacterial disease caused by infection with the intracellular pathogen *Mycobacterium tuberculosis* (Mtb), and remains the leading cause of death by a single infectious agent worldwide (1). The only approved vaccine for TB prevention is Bacille Calmette-Guérin (BCG), a live attenuated strain of *Mycobacterium bovis* (2). BCG prevents disseminated disease in infants, but its efficacy against pulmonary TB in adolescents and adults ranges from 0% to 80% across different populations (3). Despite nearly a century of use in endemic countries, BCG has not reduced the number of new cases (8 million to 10 million) reported each year (1), highlighting the need for an improved mechanistic understanding of vaccine-induced protection.

Variation in BCG’s efficacy is attributed to several factors, including geography and climate, BCG strain, and age at initial vaccination (3–6). Recently, host genetic heterogeneity has emerged as another key factor (7). While studies in C57BL/6 (B6) and other inbred strains have been invaluable in defining the immunological response to infection, there has yet to be a successful vaccine developed using the mouse model that has been effective in people. Although it is uncertain whether this shortcoming is a problem with the model or the vaccines, the reliance on inbred mice constrains our understanding of how host genetic variation modifies susceptibility to infection and shapes immune responses stimulated by vaccines.

To identify correlates of vaccine-induced protection relevant to diverse human populations (8), we turned to Collaborative Cross (CC) mice. CC mice are recombinant inbred mouse strains derived from 8 genetically distinct founder strains, developed to extend genetic mapping capabilities and develop new models that capture human phenotypes not observed in standard inbred strains (9–11). There is a wide variation in susceptibility to Mtb infection (MTBI) among different CC strains (12, 13). BCG-mediated protection among CC strains also varies with genetic background, and vaccine-induced protection is a trait that is distinct from Mtb susceptibility (10). BCG elicits a variety of immune responses among CC strains, highlighting the need to identify correlates of protection through the lens of genetic diversity (14).

CC042 mice are highly susceptible to Mtb, partly because of a splice variant in the *Itgal* gene that results in a defective CD11a protein and reduced capacity to recruit lymphocytes to the lungs following infection (15). *Itgal* encodes CD11a, the α chain (αL) of the heterodimeric αLβ2 integrin known as leukocyte functional–associated antigen-1 (LFA-1) (16). LFA-1 is an important adhesion molecule expressed on the surface of lymphocytes, and it contributes to immune cell trafficking and immune synapse formation (16–18). Similar to CC042 mice, CD11a*^–/–^* mice on the B6 genetic background are also more susceptible to Mtb, consistent with a crucial role for LFA-1 in Mtb control (19).

Here, we report that despite the lack of LFA-1 and impaired T cell trafficking to the lung (10), BCG vaccination protects CC042 mice against low-dose aerosol MTBI. Given the defect in T cell recruitment to the lung, we hypothesized that BCG protects CC042 mice by a T cell–independent mechanism. A priori, such a mechanism could be unique to CC042 mice, or the *Itgal* mutation might reveal mechanisms of BCG-mediated protection that are masked in other mouse strains. Thus, we surmised that understanding the mechanism of BCG-mediated protection in CC042 mice would reveal LFA-1–independent mechanisms of BCG-induced immunity. Here we report that lymphocyte trafficking to the lung is not necessary for BCG-mediated protection of CC042 mice 4 weeks after MTBI. In fact, depletion of memory T cell or B cell responses did not affect short-term protection against Mtb. However, optimal survival requires memory T cells. Thus, CC042 mice reveal that BCG-induced protection against low-dose aerosol MTBI at the standard time point of 4 weeks requires T cells to be present at the time of vaccination but is not mediated by conventional CD4^+^ or CD8^+^ T cells, or B cells. In contrast, memory T cells are crucial for long-term survival conferred by BCG vaccination. Our results provide insight into the mechanisms of BCG-elicited immune protection and establish CC042 mice as a new model for preclinical vaccine testing.

## Results

### BCG vaccination protects CC042 mice against Mtb challenge.

**Figure 1:**
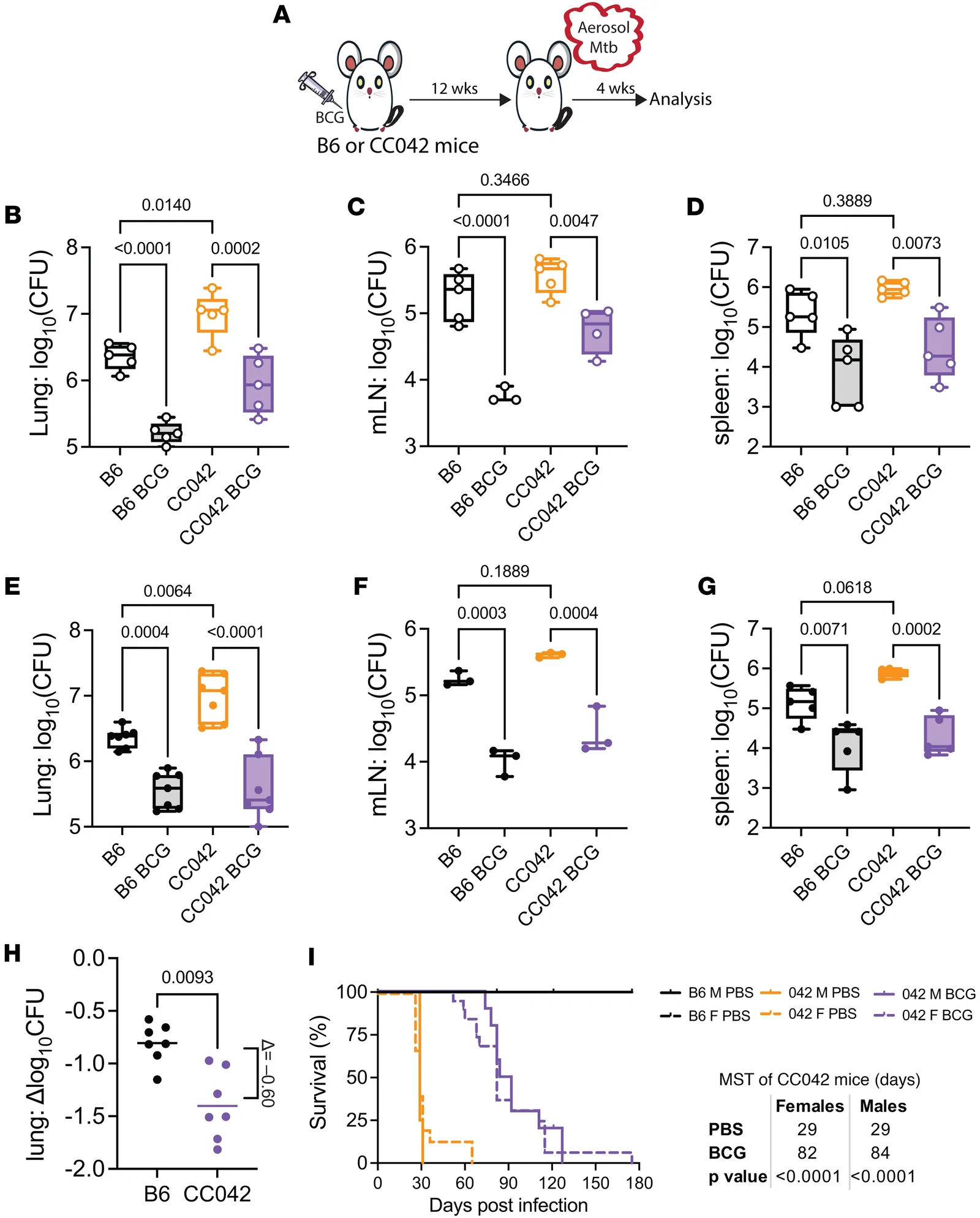
BCG protects CC042 mice against TB. (**A**) Experimental scheme. B6 and CC042 mice were vaccinated or not vaccinated with BCG in the flank and rested for 12 weeks. Then, all mice were infected with Rv.YFP and analyzed after 4 weeks. (**B**–**D**) Representative experiment showing CFU in the lung (**B**), mLN (**C**), and spleen (**D**); *n* = 4–5 mice per group. Each point is an individual mouse. (**E**–**G**) Results combined from 7 (lung; **E**), 3 (mLN; **F**), and 5 (spleen; **G**) experiments. Each point is the mean of an independent experiment. (**H**) CFU reduction in the lung from 7 experiments. Δlog_10_CFU = log_10_CFU (BCG) – log_10_CFU (unvaccinated). Each point represents an independent experiment. (**I**) Cumulative survival of non-vaccinated B6, non-vaccinated CC042, and BCG-vaccinated mice after aerosolized Mtb Erdman infection. Data compiled from 3 independent experiments; 15 B6 female mice, 10 B6 male mice, 15 CC042 female mice, 12 CC042 male mice, 19 CC042 female BCG-vaccinated mice, 11 CC042 male BCG-vaccinated mice. The difference between non-vaccinated B6 and non-vaccinated CC042 and the difference between non-vaccinated CC042 and BCG-vaccinated CC042 are statistically significant (*P* < 0.0001). (**B**–**G**) One-way ANOVA with Šidák’s multiple-comparison test. Box-and-whisker plots indicate median (middle line), 25th and 75th percentiles (box), and minimum and maximum (whiskers). (**H**) Paired *t* test. Line, mean. (**I**) Mantel-Cox log-rank test.

To confirm the results from our prior screen (10), B6 and CC042 mice were vaccinated subcutaneously with BCG or left unvaccinated. After 12 weeks of rest, all mice were challenged with low-dose aerosolized Mtb (strain Rv.YFP) for 4 weeks (Figure 1A). BCG vaccination significantly reduced Mtb burdens in the lung, mediastinal lymph node (mLN), and spleen in B6 and CC042 mice compared with unvaccinated controls (Figure 1, B–D). These results were consistent across multiple experiments (Figure 1, E–G). Interestingly, the magnitude of protection (i.e., net reduction of Mtb CFU in vaccinated vs. unvaccinated mice) was greater in CC042 mice than in B6 mice, which is typically a 10-fold reduction (Figure 1H). Consistent with previous findings, CC042 mice were substantially more susceptible to MTBI with significantly greater lung Mtb burdens than B6 mice (Figure 1B). The Mtb burdens in the mLN and spleen were also greater in CC042 mice than in B6 mice, but this did not reach statistical significance (Figure 1, C and D).

We next tested whether BCG vaccination improves survival of CC042 mice following MTBI. BCG-vaccinated and unvaccinated control CC042 mice were rested for 12 weeks and then challenged with low-dose aerosolized Mtb Erdman. Following infection, unvaccinated B6 mice survived beyond 180 days, while the unvaccinated CC042 mice had a median survival of 29 days (Figure 1I). BCG vaccination extended the median survival of Mtb-infected CC042 mice to 82–88 days and provided comparable benefit to both sexes. These data show that BCG confers durable protection to CC042 mice despite their lack of CD11a.

### BCG vaccination promotes lymphocyte infiltration into the lungs during MTBI.

**Figure 2:**
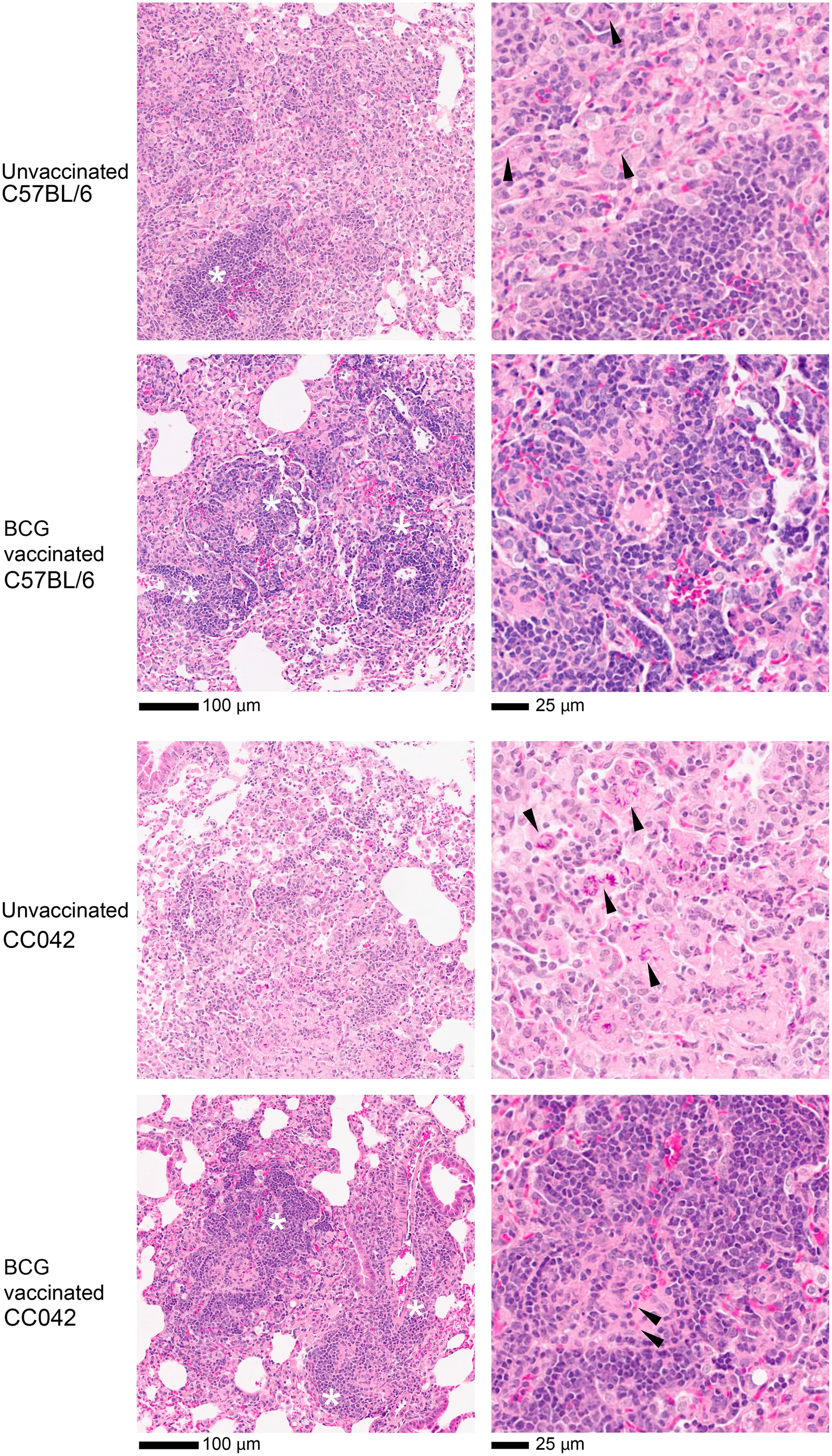
BCG reduces the severity of disease in the lungs of CC042 mice. The caudal lung lobes from unvaccinated or BCG-vaccinated mice were obtained 4 weeks after Mtb challenge, and sections were stained with a combined AFB and H&E stain. The first and second rows are images from unvaccinated and BCG-vaccinated B6 mice, respectively. The third and fourth rows are images from unvaccinated and BCG-vaccinated CC042 mice, respectively. BCG was given in the flank. Scale bars: 100 μm (left panels) and 25 μm (right panels). Each image is representative of an individual subject (*n* = 4–5 per group), and the images are from 1 of 3 independent experiments with similar results. Black arrowheads, AFB (although not all AFB are identified with arrowheads); white asterisks, lymphocyte.

To understand how BCG protects CC042 mice, caudal lung lobes were collected 4 weeks post-infection (wpi) (Figure 1A), fixed in formalin, and stained with a combined acid-fast bacterial (AFB) stain plus hematoxylin and eosin (H&E) (Figure 2). Unvaccinated B6 mice had mild to moderate disease with circumscribed lesions containing well-defined lymphocytic infiltrates and few AFB, which, when detected, were mostly seen as single bacilli (Figure 2, first row). BCG-vaccinated B6 mice had mild disease with prominent lymphocyte cuffing and dense lymphocytic infiltrates, few foamy macrophages with small foci of neutrophils, and rare AFB (Figure 2, second row). In contrast, CC042 mice had mild to moderate disease with diffusely organized lymphohistiocytic lesions and abundant AFB clusters (Figure 2, third row). The lesions in BCG-vaccinated CC042 mice were similar to the B6 lesions: smaller and well-defined, containing more lymphocytic infiltrates than in unvaccinated mice and fewer AFB (Figure 2, fourth row). Overall, in the unvaccinated state, susceptible CC042 mice had disorganized, loosely structured, lymphocyte-poor lung lesions with poor control of Mtb replication. BCG vaccination of CC042 mice led to compact lesions with more lymphocytes and fewer bacilli.

### BCG-mediated protection of CC042 mice does not require lymphocyte trafficking to the lung.

**Figure 3:**
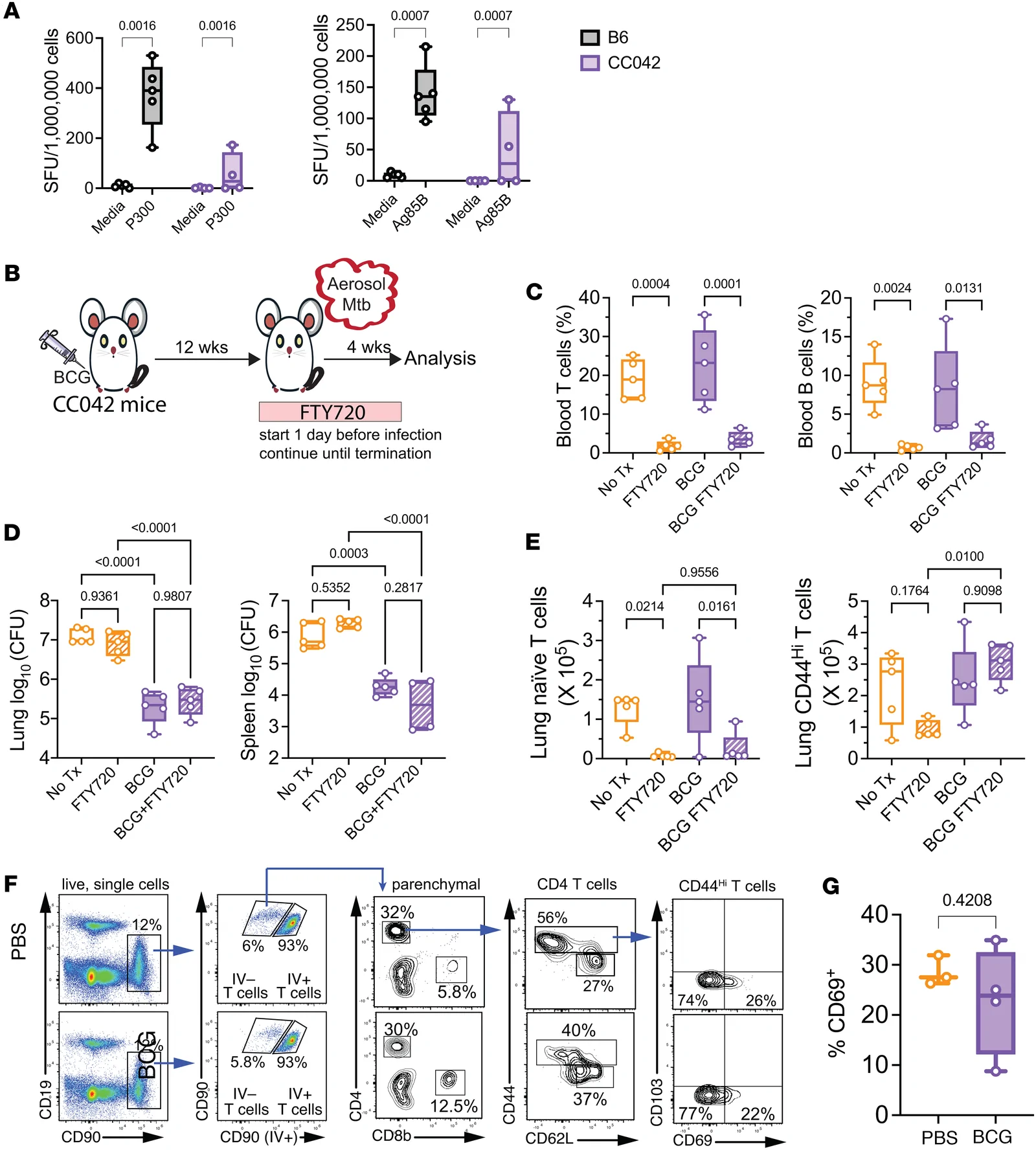
Protection of CC042 mice by BCG is not abrogated by FTY720 treatment. (**A**) The number of IFN-γ spot-forming units (SFU) per million popliteal lymph node (pLN) cells of BCG-vaccinated B6 and CC042 mice after incubation with the P300 peptide pool (left) or peptide Ag85B (right) was determined following 44-hour coculture via ELISPOT. Each dot represents 1 subject; *n* = 4–5 mice per strain. Data are from 1 of 2 independent experiments with similar results. (**B**) Experimental scheme. CC042 mice were vaccinated or not vaccinated in the flank with BCG and rested for 12 weeks. FTY720 (4 mg/kg) was administered to half the mice in each group, starting 1 day before infection and continuing for 4 weeks. All mice were infected with Rv.YFP and analyzed at 4 wpi. (**C**) Analysis of FTY720-treated CC042 mice. Frequency of T and B cells, as a percentage of total live cells, in blood of different groups of control and vaccinated mice, untreated or treated with FTY720. (**D**) CFU in lung (left) and spleen (right) of CC042 mice at 4 wpi. (**E**) Naive (left, CD44^–^CD62L^+^) and antigen-experienced (right, CD44^+^CD62L^–^) T cell counts in lungs of CC042 mice at 4 wpi. (**F**) Analysis of T cells from lungs of CC042 mice, 12 weeks after BCG vaccination. Control (top row) or BCG-vaccinated (bottom row) mice were injected i.v. with anti-CD90–AF647, euthanized, and analyzed by flow cytometry. First plot is gated by scatter, live cells, and CD90, and shows CD4 expression of cells in the vasculature (IV^+^) versus in the parenchyma (IV^–^). The second plot shows CD44 and CD69 expression of cells that are CD4^+^IV^+^. (**G**) Lung parenchymal CD4^+^CD44^hi^ T cells with a Trm phenotype (CD69^+^) were enumerated 12 weeks after vaccination with or without BCG. (**A**, **C**–**E**, and **G**) Two-way ANOVA with Šidák’s multiple-comparison test (**A** and **C**–**E**) or *t* test (**G**). Box-and-whisker plots indicate median (middle line), 25th and 75th percentiles (box), and minimum and maximum (whiskers). Each point represents an individual subject; *n* = 3–5 mice per group. Data are from 1 of 2 independent experiments with similar results.

We were surprised to detect more lymphocytic infiltrates in BCG-vaccinated CC042 mice (Figure 2), as few ESAT6 or TB10.4 tetramer–positive T cells are detected in their lungs after primary MTBI, presumably because of CD11a loss (15). CD11a is critical for naive T cell trafficking into LNs, and we previously showed that priming and lung recruitment of ESAT6-specific CD4^+^ T cells are impaired after MTBI in CD11a-knockout mice (19, 20). To assess T cell responses in CC042 mice following BCG vaccination, we used Ag85B/I-A^b^ and TB10.4/K^b^ tetramers, intracellular cytokine staining, and ELISPOT assays. Because BCG-specific T cell responses after vaccination at the flank were below the limit of detection, we used vaccinated mice at the hock and measured T cell responses in the popliteal LN (21). We detected responses to MTB300 and Ag85B, although there was considerable variability in CC042 responses, which were considerably weaker than those in B6 control mice (Figure 3A).

We hypothesized that BCG-induced T cells traffic to the lung independently of CD11a. To test this hypothesis, we disrupted lymphocyte trafficking by treating mice with FTY720, which degrades the sphingosine 1-phosphate receptor and prevents lymphocyte egress from LNs (22). Dose optimization in B6 mice showed that 1 mg/kg and 4 mg/kg reduced lymphocyte numbers in blood and in lungs (Supplemental Figure 1, A–C; supplemental material available online with this article; https://doi.org/10.1172/jci.insight.201000DS1), but only high-dose FTY720 increased lung Mtb burden in B6 mice (Supplemental Figure 1D). Therefore, 4 mg/kg was used in subsequent experiments.

CC042 and B6 mice were vaccinated with BCG, or left unvaccinated as controls, and all mice were rested for 12 weeks. All mice were treated with FTY720 commencing the day before low-dose aerosolized Mtb Rv.YFP infection (Figure 3B). FTY720 treatment reduced circulating T and B cells in both unvaccinated and vaccinated CC042 mice by 83%–93% during infection (Figure 3C; and gating scheme, Supplemental Figure 2). As expected, FTY720 treatment of unvaccinated B6 mice led to an increase in Mtb CFU in the lung (Supplemental Figure 1E). Surprisingly, FTY720 treatment of unvaccinated CC042 mice had no effect on lung or spleen Mtb CFU, nor did it impair BCG-induced protection of CC042 mice (Figure 3D).

BCG vaccination generates lung-resident memory T cells (Trms) in both humans and mouse models, which are implicated in protection against TB (18, 23–26). Since BCG reduced lung CFU at 4 wpi, even when lymphocyte trafficking was blocked, we considered whether BCG elicited Trms. In both BCG-vaccinated and unvaccinated CC042 mice, FTY720 significantly (85%–94%) reduced naive T cells in the lung (Figure 3E, left; and gating scheme, Supplemental Figure 3). FTY720 also reduced the number of antigen-experienced CD44^hi^ T cells by 71% in unvaccinated CC042 mice. However, the CD44^hi^ T cells in the lungs of vaccinated CC042 mice were resistant to FTY720, meaning that despite FTY720 treatment, a prominent population of CD44^hi^ T cells were found in the lungs of Mtb-infected mice (Figure 3E, right). As these data were consistent with a Trm population, we analyzed the lungs 12 weeks after BCG vaccination and before Mtb challenge. Few parenchymal T cells and no significant increase in Trms (CD4^+^CD44^hi^CD69^+^) were detected after vaccination and prior to infection (Figure 3, F and G). Although some Trms may not be released by enzymatic digestion of lung tissue, these findings suggest that BCG-mediated protection in CC042 mice is not leading to the establishment of CD4^+^ or CD8^+^ Trms in the lung prior to infection.

### Bacillary control in BCG-vaccinated CC042 mice challenged with Mtb is independent of CD4^+^ and CD8^+^ memory T cells.

**Figure 4:**
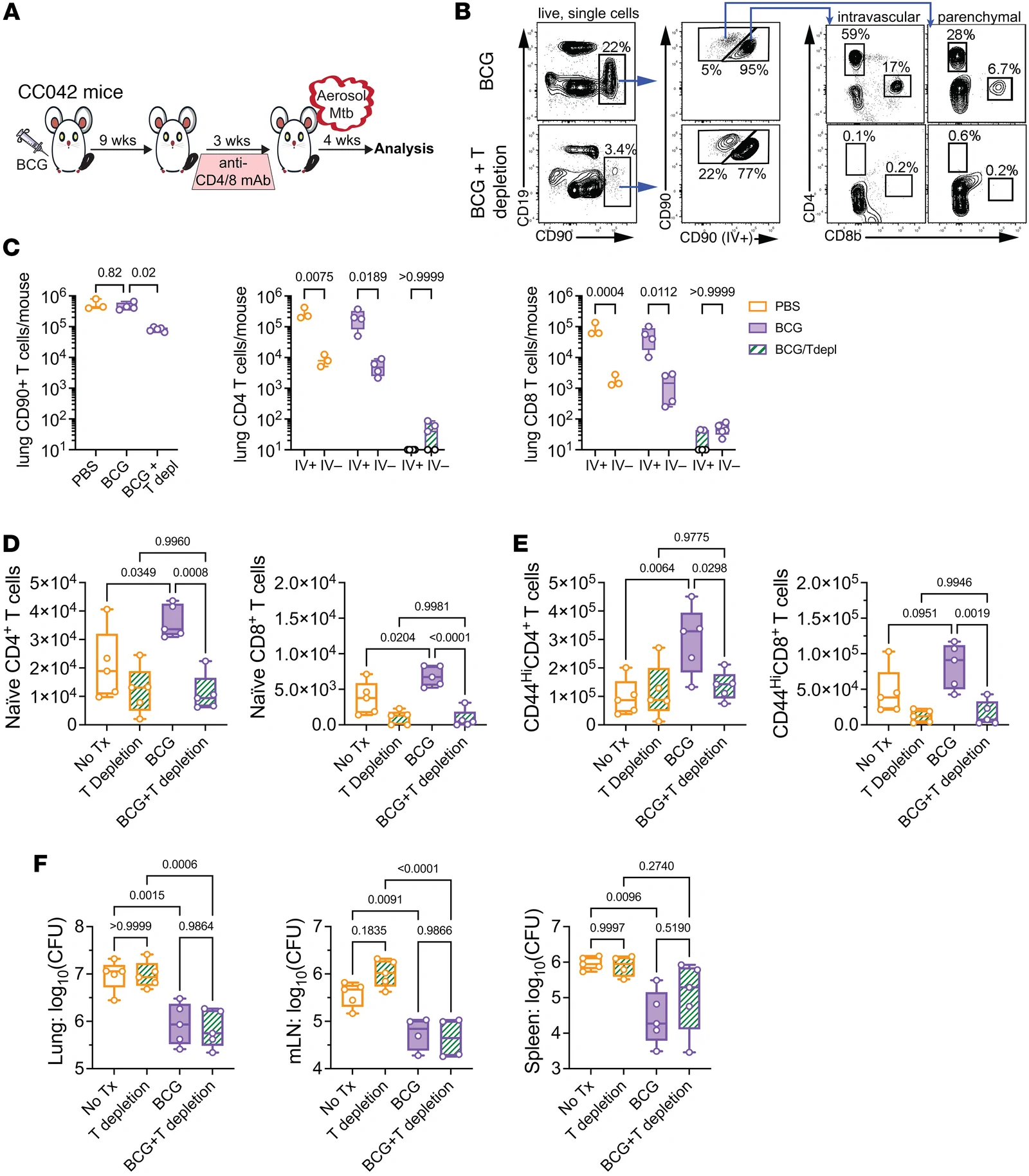
Bacterial control in BCG-vaccinated CC042 mice challenged with Mtb is independent of CD4^+^ and CD8^+^ memory T cells. (**A**) Experimental scheme. CC042 mice were vaccinated or not vaccinated with BCG in the flank and rested for 9 weeks. A cohort from each vaccination group was treated with a combination of anti-CD4 and anti-CD8α mAbs for 3 weeks. Then, all mice were infected for 4 weeks with Rv.YFP. (**B**) Determination of CD4^+^ and CD8^+^ T cell depletion. Mice were treated with anti-CD4 and anti-CD8α mAbs after BCG vaccination as described in **A**. The location of the CD4^+^ and CD8β^+^ T cells was determined as described in Methods, and the populations are designated as either IV^+^ (intravascular) or IV^–^ (parenchymal). Representative flow plot of CD4 and CD8β expression of CD90^+^ T cells in the blood at 4 wpi of representative BCG-vaccinated CC042 mice with and without T depletion. These data are from the same experiment depicted in Figure 3F. (**C**) Frequency of IV^+^ or IV^–^ CD90^+^, CD4^+^, and CD8β^+^ T cells in the lung at the end of the depletion period but before MTBI of CC042 mice. (**D** and **E**) Enumeration of naive (CD44^–^CD62L^+^, **D**) and antigen-experienced (CD44^+^CD62L^–^, **E**) CD4^+^ (left) and CD8^+^ (right) T cells in the lungs of CC042 mice 4 weeks after infection. (**F**) BCG-elicited protection in the lung (left), mLN (middle), and spleen (right) is not affected by CD4^+^ and CD8^+^ T cell depletion. (**C**–**F**) One-way ANOVA with Šidák’s multiple-comparison test. Box-and-whisker plots indicate median (middle line), 25th and 75th percentiles (box), and minimum and maximum (whiskers). Each point represents an individual subject; *n* = 3–5 mice per group. The data are from 1 of 2 independent experiments with similar results.

To determine whether BCG protection of CC042 mice is mediated by conventional memory T cell responses, we transiently depleted T cells using anti-CD4/CD8α mAb for 3 weeks starting 9 weeks after vaccination (Figure 4A). By depleting T cells after BCG and before MTBI, we expected that naive T cell replenishment would occur with the net effect of preferentially depleting memory T cells and abrogating T cell recall responses. We confirmed that the depleting antibody does not interfere with the analysis of depletion efficiency (Supplemental Figure 4A). The antibody cocktail eliminated 99% and 94% of circulating CD4^+^ and CD8^+^ T cells, respectively (Supplemental Figure 4B), and significantly reduced T cells in inguinal lymph node (ILN), lung, and spleen (Supplemental Figure 4, C and D). NK cells were unaffected (Supplemental Figure 4D). To assess depletion of lung parenchymal T cells, mice were given intravenous anti-CD90 (see Methods) before euthanasia to label intravascular T cells in the lung (25). Anti-CD4/8 treatment eliminated nearly all intravascular and parenchymal T cells (Figure 4, B and C). The remaining cells were CD4^–^CD8^–^ T cells (Figure 4B) and mostly γδ T cells.

Given the difficulty in detecting antigen-specific T cell responses in these mice (Figure 3A), presumably due to poor naive T cell localization in LNs, poor priming, and delayed recruitment to the lung (19), we identified naive cells (CD62L^+^CD44^lo^) and antigen-experienced (CD62L^–^CD44^hi^) T cells. Four weeks after Mtb challenge, BCG-vaccinated mice had an increase in naive CD4^+^ and CD8^+^ T cells and a more significant increase of antigen-experienced CD4^+^ T cells (~3-fold, *P* = 0.0098) (Figure 4, D and E; and gating scheme, Supplemental Figure 3) compared with unvaccinated controls. Importantly, BCG vaccination led to about 10-fold more CD44^hi^ T cells than naive T cells (Figure 4, D and E). Anti-CD4/8 treatment after BCG administration prevented these expansions (Figure 4E). The number of CD44^hi^ CD4^+^ T cells in the lungs of the “BCG+T depletion” and “No Tx” groups was similar, indicating effective depletion of BCG-primed T cells.

To determine whether memory T cells were required for protection, we quantified Mtb in lung, mLN, and spleen. T cell depletion prior to challenge did not affect Mtb burden in unvaccinated mice (“No Tx” vs. “T depletion,” Figure 4F), presumably because naive CD4^+^ T cells are replenished before the endpoint, and few T cells reach the lung even in undepleted mice because of the CD11a deficiency (15). Interestingly, BCG-mediated protection was maintained in the lungs, mLNs, and spleens of CC042 mice despite CD4^+^ and CD8^+^ T cell depletion (“BCG” vs. “BCG+T depletion,” Figure 4F). Thus, conventional memory CD4^+^ and CD8^+^ T cells do not appear to mediate protection in BCG-vaccinated CC042 mice at 4 wpi. Instead, early vaccine-mediated protection of CC042 mice may be mediated by another mechanism.

### B cells are not required for BCG-mediated protection of CC042 mice.

**Figure 5:**
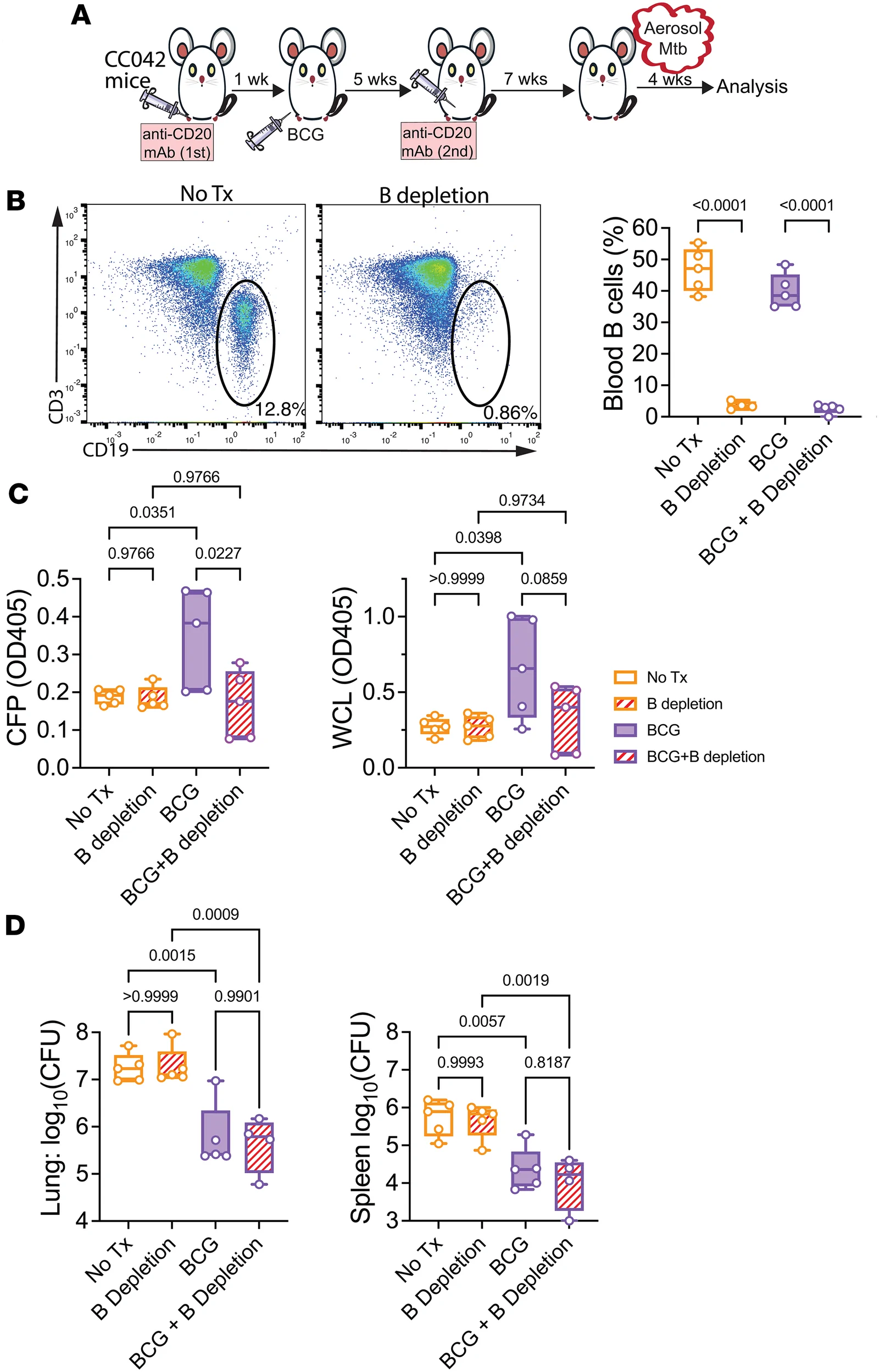
Depletion of B cells does not abrogate BCG-mediated protection in CC042 mice. (**A**) Experimental scheme. CC042 mice were treated with anti-CD20 depletion mAbs 1 day before and 5 weeks after BCG vaccination in the flank. Mice were rested for 12 weeks after vaccination, and then all mice were infected with Rv.YFP for 4 weeks. (**B**) Representative flow plots (left) showing CD19 (B cells) and CD3 (T cells) expression of total live cells in the blood of BCG-vaccinated versus BCG-vaccinated and B cell–depleted CC042 mice and frequency (right) of CD19^+^ B cells in blood 4 weeks after BCG vaccination of CC042 mice, as determined by flow cytometry. (**C**) Quantification of total serum antibodies specific to CFP (left) and WCL (right) at the endpoint. Each point represents the mean of technical duplicate replicates; *n* = 5 mice per group. (**D**) CFU in the lung (left) and spleen (right) at 4 wpi. One-way ANOVA with Šidák’s multiple-comparison test. Each point represents an individual subject; *n* = 4–5 mice per group. (**B**–**D**) Data are from 1 of 2 independent experiments with similar results. One-way ANOVA with Šidák’s multiple-comparison test. Each point represents an individual subject; *n* = 5 mice per group. Box-and-whisker plots indicate median (middle line), 25th and 75th percentiles (box), and minimum and maximum (whiskers).

As BCG stimulated short-term protection by a seemingly T cell–independent mechanism, we hypothesized that B cells or antibody responses were responsible for CFU at 4 wpi. Anti-CD20 mAb was administered to deplete B cells 1 week before BCG vaccination to prevent initiation of antibody responses to mycobacterial antigens, and a second dose was given 5 weeks after vaccination (Figure 5A) (27). The depleting anti-CD20 mAb antibody did not interfere with B cell identification by flow cytometry (Supplemental Figure 4A), and we verified that anti-CD20 mAb treatment reduced B cells in the lung and ILN by 78% and 99%, respectively (Supplemental Figure 5, A and B). Four weeks after BCG vaccination, peripheral blood B cells were reduced by 92%–94% in comparison with untreated CC042 mice (Figure 5B).

To assess the functional consequences of B cell depletion, antibody titers to culture filtrate protein (CFP) and whole-cell lysate (WCL) were determined at 4 wpi. Although antibody levels were low and variable, BCG-vaccinated CC042 mice had an increase in anti-CFP and anti-WCL antibodies compared with unvaccinated mice, which was abrogated by B cell depletion prior to vaccination and during the rest period (Figure 5C). The Mtb burden in the lung and spleen was similarly reduced in the “BCG” and “BCG+B depletion” groups, compared with unvaccinated mice (Figure 5D). Thus, B cell and antibody responses were dispensable for protection induced by BCG vaccination in CC042 mice.

### BCG does not disseminate to the bone marrow nor alter hematopoietic stem cells after subcutaneous vaccination.

**Figure 6:**
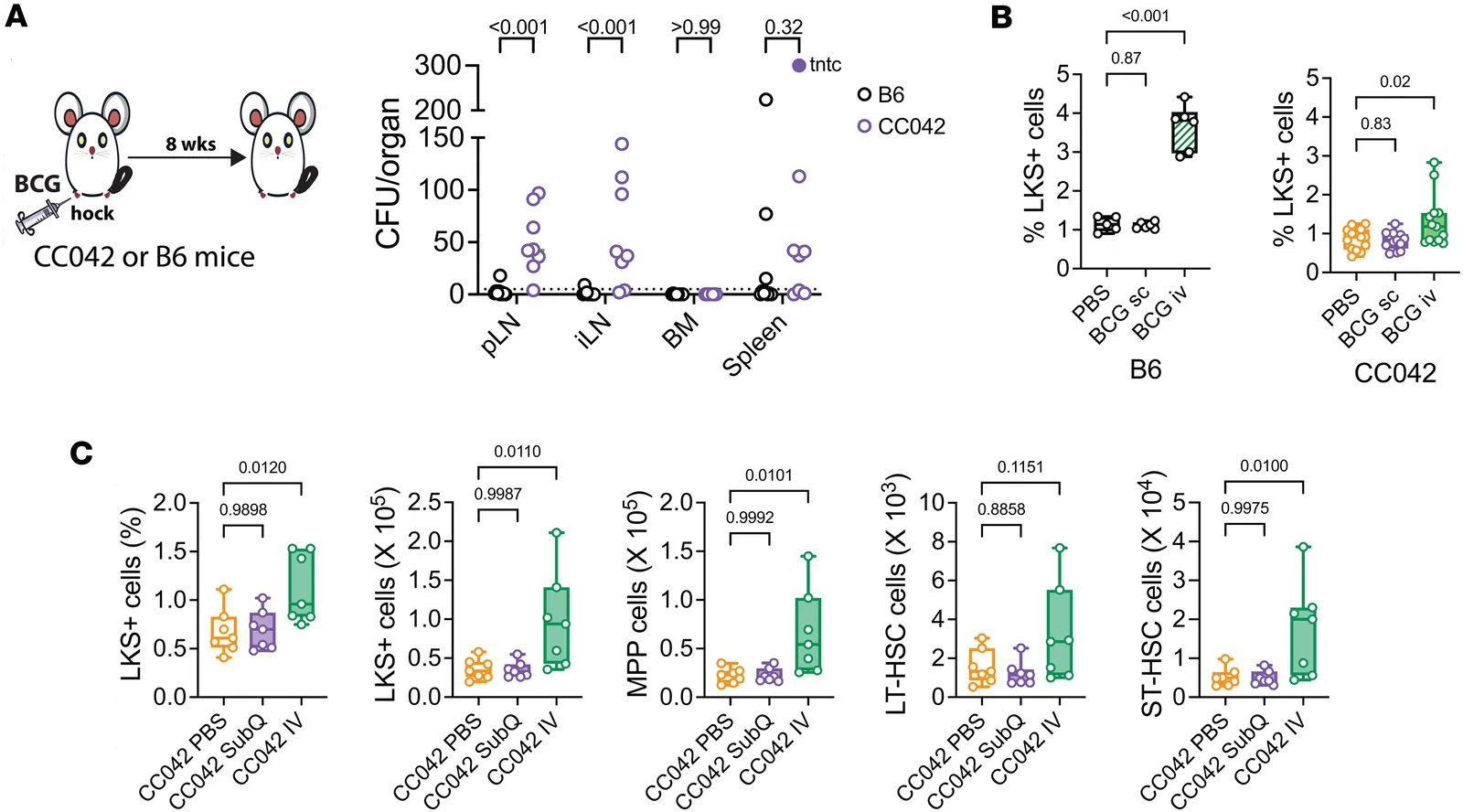
Subcutaneous BCG vaccination of CC042 mice does not result in HSC expansion in the bone marrow. (**A**) B6 and CC042 mice were vaccinated subcutaneously at the right hock, and the number of viable BCG recovered 8 weeks after vaccination per organ is plotted. BCG persists in the ipsilateral (right) popliteal lymph node (pLN), ILN, and spleen of both strains. The bone marrow (BM) is sterile in both B6 and CC042 mice 8 weeks after vaccination. Data are combined from 2 independent experiments with 4–5 mice per group. The limit of detection was 5 CFU per organ. (**B**) B6 and CC042 mice were sham vaccinated with PBS via the i.v. route or vaccinated with BCG via the i.v. or s.c. route. After 4 weeks, the percentage of LKS^+^ cells was determined. Data are pooled from 2 experiments for B6 mice (5–6 mice per group) and 3 experiments for CC042 (13 mice per group). (**C**) CC042 mice (7 mice per group) were sham vaccinated with PBS via the i.v. route or vaccinated with BCG via the i.v. or s.c. route, and the expansion of HSCs was determined 4 weeks after vaccination in the BM. Representative result of 3 independent experiments with similar results. Statistical testing was performed using the Mann-Whitney *t* test (**A**), Holm-Šidák multiple-comparison test (**B**), or Dunnett’s multiple-comparison test (**C**). Box-and-whisker plots indicate median (middle line), 25th and 75th percentiles (box), and minimum and maximum (whiskers).

BCG protection of infants from pathogens other than Mtb led to the discovery of cross-immunity and innate trained immunity (28–30). In B6 mice, intravenous BCG dissseminates to the bone marrow (BM) and induces epigenetic modifications in hematopoietic stem cells (HSCs), resulting in BM-derived macrophages with a greater capacity to restrict intracellular Mtb replication (31). As all our experiments administered BCG by the subcutaneous route, the T cell defects in CC042 mice might be unable to contain BCG. No cultivable BCG was detected in B6 or CC042 BM 8 weeks after subcutaneous vaccination. However, BCG persisted in other tissues, such as CC042 draining lymph nodes (Figure 6A).

As BCG could transiently reside in the BM, or below our detection threshold, we assessed changes among HSCs following subcutaneous or intravenous BCG. The intravenous route was a control that forces the entry of BCG into the BM (31). Intravenous BCG expanded the frequency of BM Lin^–^c-Kit^+^Sca-1^+^ (LKS^+^) cells in B6 and CC042 mice, compared with subcutaneous BCG or PBS-treated controls (Figure 6B). The effects of intravenous and subcutaneous BCG vaccination on the BM compartment of CC042 mice were investigated further. There were no differences in the frequency or number of LKS^+^ cells, multipotent progenitors (MPPs), short-term HSCs (ST-HSCs), and long-term HSCs (LT-HSCs) between subcutaneous-BCG-vaccinated and PBS-treated CC042 mice (Figure 6C; and gating scheme, Supplemental Figure 6). Although we cannot rule out the possibility that subcutaneous BCG vaccination altered HSCs at another time point, we conclude that it is unlikely that innate training through HSC remodeling is how BCG mediates short-term protection against Mtb in CC042 mice.

### T cell responses after BCG vaccination help prime innate-like protection against Mtb.

**Figure 7:**
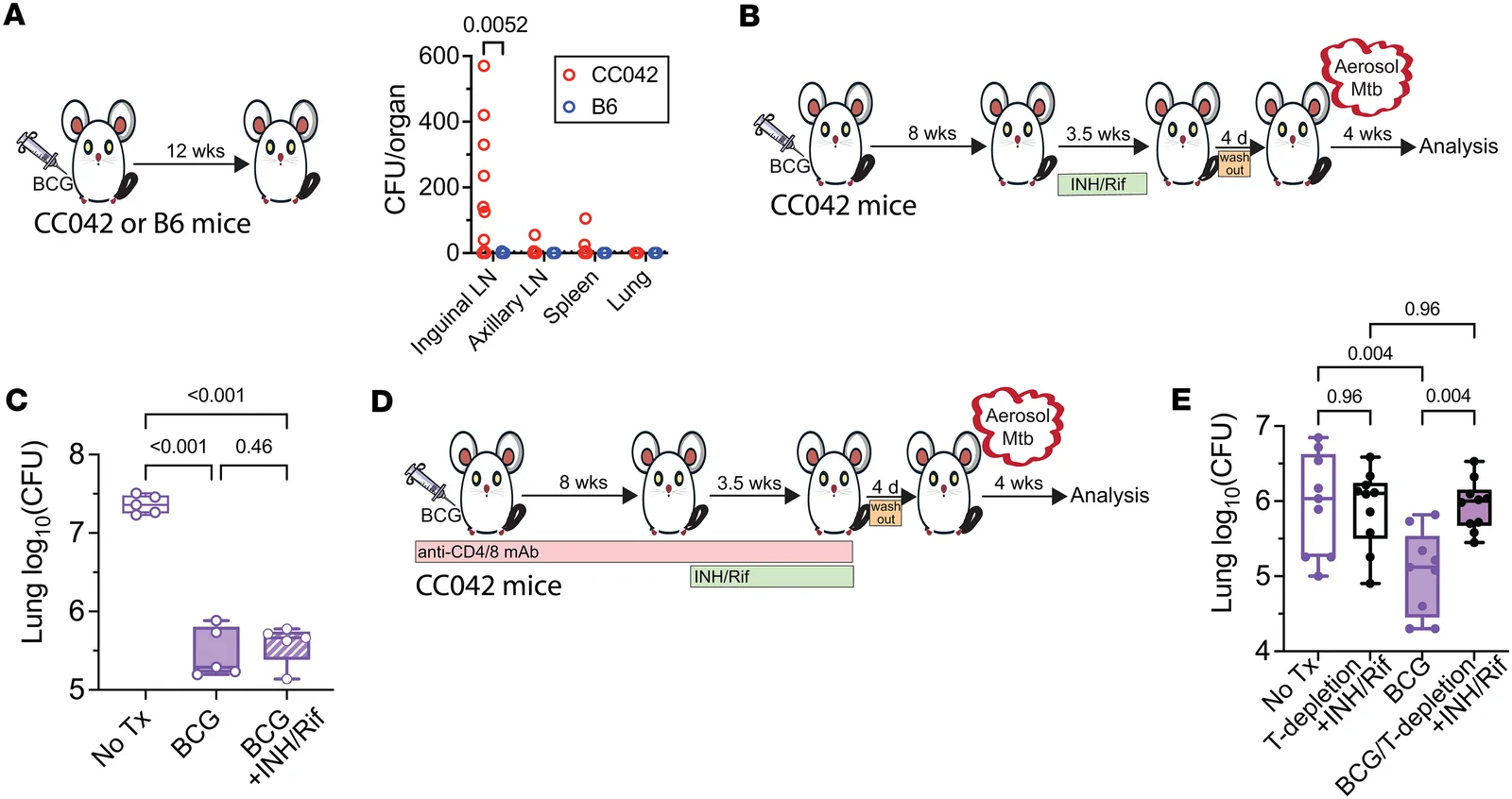
The presence of T cells during vaccination is required for the development of protective immunity. (**A**) B6 and CC042 mice were vaccinated with BCG in the flank, and 12 weeks later, the presence of viable BCG was enumerated in the ILN, axillary LN, spleen, and lung. Data are pooled from results of 2 independent experiments; *n* = 10 mice per group. The limit of detection was 5 CFU. (**B**) Experimental scheme. CC042 mice were vaccinated or not vaccinated with s.c. BCG in the flank and then rested for 8 weeks. Half of the BCG-vaccinated mice were then provided with drinking water with both isoniazid and rifampicin (INH/Rif) for 3.5 weeks. Twelve weeks after BCG vaccination, all mice were infected with Rv.YFP for 4 weeks. (**C**) CFU in the lung. (**D**) Experimental scheme. CC042 mice were treated with CD4 and CD8α depletion antibodies starting 1 week before BCG vaccination and for the duration of the 12-week rest period. Nine weeks after vaccination, all mice within the depletion group were provided with drinking water with INH/Rif for 2.5 weeks. Twelve weeks after BCG vaccination, all mice were infected with Rv.YFP for 4 weeks. (**E**) CFU in the lung. Statistical testing was performed using the Mann-Whitney *t* test (**A**) or a 1-way ANOVA with Šidák’s multiple-comparison test (**C** and **E**). Each point represents an individual subject; *n* = 4–5 mice per group. Data are representative of 2 independent experiments with similar results.

Given the persistence of BCG in CC042 mice after subcutaneous vaccination (Figure 6A), we next looked at the 12-week time point, the end of the vaccine rest period. Three of ten B6 mice had 5–20 bacilli in their ILNs, but no BCG was detected in the non-draining LN, spleen, or lung (Figure 7A). In contrast, BCG was detected in the spleen and LN of CC042 mice, with 8 of 10 mice having bacilli in the ILN, and 6 having more than 125 CFU (Figure 7A). We wondered whether persistent BCG could lead to chronic immune activation and short-term protection of CC042 mice from MTBI.

To test this possibility, in addition to BCG-vaccinated and unvaccinated CC042 mice, a third BCG-vaccinated group was treated with isoniazid and rifampicin starting 8 weeks after vaccination to eliminate persistent BCG. Eight weeks was chosen to permit the immune responses to evolve but ensure sufficient time to kill any persistent BCG. After 3.5 weeks of antibiotic treatment, all viable BCG were eliminated from the ILN of vaccinated CC042 mice (Supplemental Figure 7A). The mice were given a 4-day washout period and infected with low-dose aerosolized Rv.YFP for 4 weeks, and lung CFU were assessed (Figure 7B). Elimination of persistent BCG following vaccination does not impair protection in CC042 mice, as the bacterial burdens in the lungs of the “BCG” and “BCG/INH+Rif (isoniazid and rifampicin)” (BCG+abx) groups were not significantly different from each other and were significantly reduced in comparison with the unvaccinated group (“No Tx”) (Figure 7C). These data show that viable BCG remaining in the ILN are not required to protect CC042 mice.

While memory CD4^+^ and CD8^+^ T cells do not appear to mediate the effector phase of BCG-elicited protection against MTBI (Figure 4), we asked whether BCG-induced protection requires T cells during the initiation of the vaccine response. Instead of depleting CD4^+^ and CD8^+^ T cells 3 weeks before MTBI, we depleted CD4^+^ and CD8^+^ T cells starting 1 week before BCG vaccination and continued through the rest period (Figure 7D). As we expected BCG to persist in the absence of T cells, we treated vaccinated mice with antibiotics. After a 4-day washout period, the mice were infected with low-dose aerosolized Rv.YFP, and after 4 weeks, lung CFU were measured (Figure 7D). Sustained treatment with anti-CD4/8 mAbs resulted in depletion of 99% of circulating CD4^+^ and CD8^+^ T cells before Mtb challenge (Supplemental Figure 7B) and abrogated short-term protection in the lung (Figure 7E). Analysis of lung T cells at this time point revealed that TCRβ^+^ T cells were the major population of lung T cells; mucosal-associated invariant T cells (MAIT cells), NKT cells, and γδ T cells were minor populations (Supplemental Figure 8A). We next assessed the abundance of major T cell populations found in the lung parenchymal compartment by excluding intravascular cells (Supplemental Figure 8B). Only CD4^+^ T cells were significantly increased in the lung parenchyma during MTBI, in comparison with unvaccinated mice. Importantly, depletion of CD4^+^ and CD8^+^ T cells before Mtb challenge significantly blunted this increase (Supplemental Figure 8C). These data contrast with our earlier experiments (Figure 4), in which conventional CD4^+^ and CD8^+^ T cells were dispensable for BCG-mediated protection. Thus, while the enhanced capacity of BCG-vaccinated mice to restrict Mtb replication in the lung is not mediated by memory CD4^+^ and CD8^+^ T cells, CD4^+^ and CD8^+^ T cells are nonetheless required during the response to BCG, possibly to prime innate-like protection against Mtb challenge.

### Long-term survival of BCG-vaccinated CC042 mice challenged with Mtb requires T cells.

**Figure 8:**
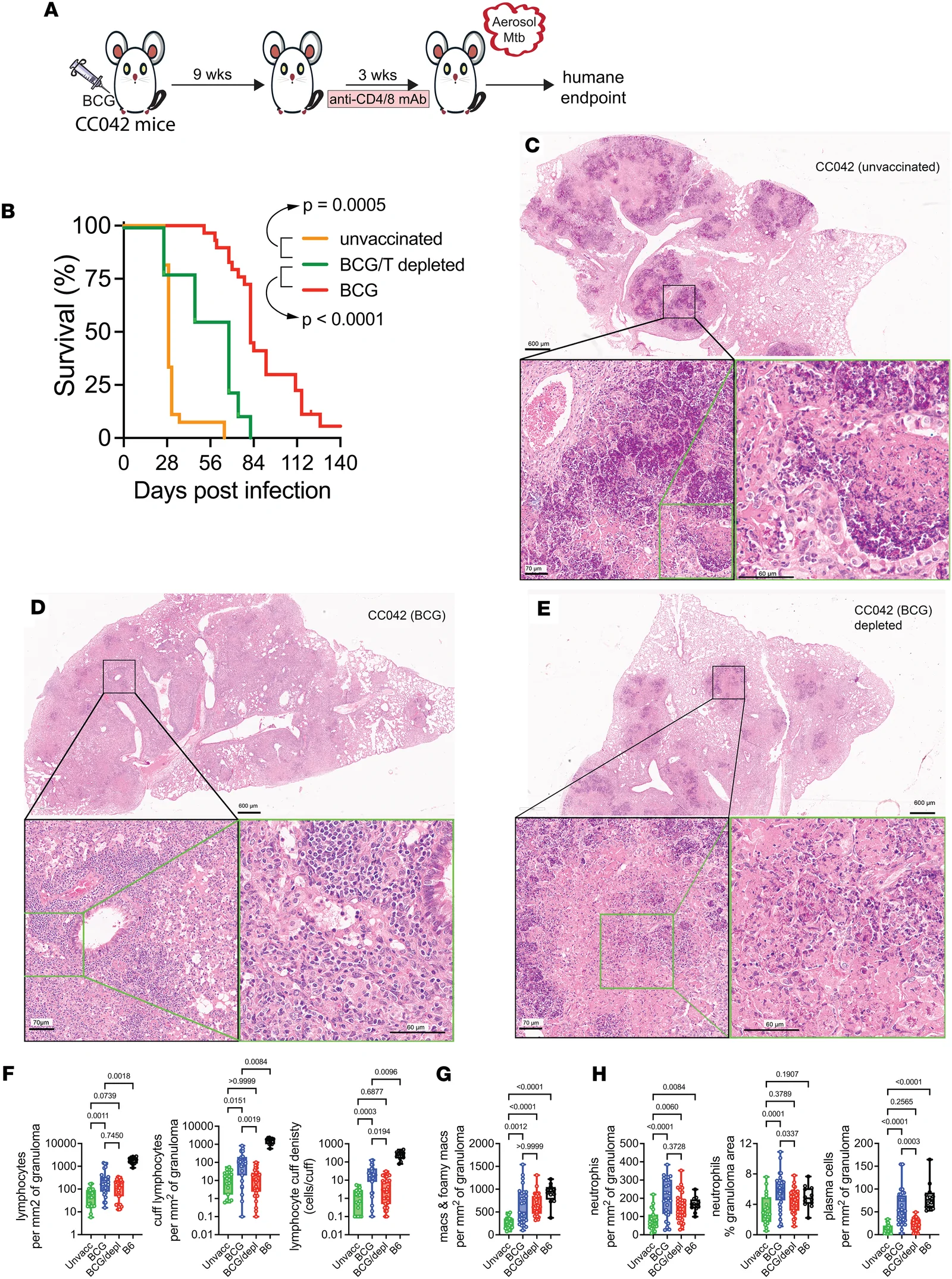
Long-term survival of BCG-vaccinated CC042 mice challenged with Mtb requires T cells. (**A**) Experimental scheme. CC042 mice were vaccinated or not vaccinated with BCG in the flank and rested for 9 weeks. Then, half of the BCG-vaccinated mice were treated with a combination of anti-CD4 and -CD8α depleting antibodies for 3 weeks. Then all mice were infected for 4 weeks with Mtb Erdman. (**B**) Unvaccinated (*n* = 27), BCG-vaccinated (*n* = 30), and BCG-vaccinated plus CD4 + CD8α–depleted (*n* = 9) mice were monitored until they reached a predetermined humane endpoint and were euthanized. Data are pooled from 3 experiments. The difference between non-vaccinated and BCG-vaccinated CC042 mice reached statistical significance based on the Mantel-Cox log-rank test (*P* < 0.0001). The difference between non-vaccinated and BCG-vaccinated and treated CC042 mice reached statistical significance based on the Mantel-Cox log-rank test (*P* = 0.0005). (**C**–**E**) Representative images of lung pathology at the time of death from an unvaccinated CC042 mouse (**C**), a BCG-vaccinated CC042 mouse (**D**), and a BCG-vaccinated and T cell–depleted CC042 mouse (**E**). Green and black boxes denote areas of higher magnification within the same image. Scale bars: 600 μm (top images), 70 μm (left magnified images), and 60 μm (right magnified images). (**F** and **G**) Automated image analysis of histopathological tissue. Three to 5 lung lobes were analyzed per mouse for a total of 19 (unvaccinated), 27 (BCG), 34 (BCG + T depletion), and 12 (B6) lobes. To account for variability in the tissue sections, the analysis was normalized to the granuloma area. Comparisons between different groups were analyzed using a non-parametric 1-way ANOVA (Kruskal-Wallis). (**F**) Total number of lymphocytes in the lesions (left); number of lymphocytes in cuff regions (middle); and density of lymphocytes per cuff (right). (**G**) Total number of granuloma macrophages. (**H**) Total number of neutrophils in the lesions (left); percentage of the granuloma area occupied by neutrophil infiltrates (middle); and number of plasma cells.

BCG vaccination generates protective immunity in CC042 mice, as defined by lung CFU reduction at the standard time point of 4 wpi, in a manner that is dependent on CD4^+^ and CD8^+^ T cells, but not mediated by memory T cell responses (Figures 1, 4, and 7). As BCG vaccination of CC042 mice extends survival (Figure 1), we considered whether protection at the early and late phases of infection was mediated by different mechanisms. We hypothesized that innate mechanisms of protection limit bacterial growth early after infection, while T cells mediate long-term protection. To test this possibility, BCG-vaccinated CC042 mice were depleted of T cells during the vaccine rest period, challenged with Mtb, and monitored for survival using weight loss as a humane endpoint (Figure 8A). We found significant depletion of total (77%), CD4^+^ (96%), and CD8^+^ (99%) T cells in the peripheral blood of BCG-vaccinated CC042 mice before Mtb challenge (Supplemental Figure 7C).

As previously observed (Figure 1I), non-vaccinated CC042 mice rapidly succumb to pulmonary TB, and BCG vaccination significantly extends survival. Depletion of CD4^+^ and CD8^+^ T cells after vaccination, but before MTBI, significantly reduced the median survival from 82 days to 68 days (*P* < 0.0001; Figure 8B), indicating that BCG-induced memory T cells mediate long-term protection of CC042 mice. Conversely, despite memory T cell depletion before Mtb challenge, the median survival was significantly increased from 28 days to 68 days, suggesting that an unknown component of long-term protection by BCG is independent of vaccine-induced T cell recall responses (*P* = 0.0005; Figure 8B).

To understand how BCG-induced memory T cells modify host immunity and TB pathogenesis, we examined lung tissue collected at the time of euthanasia from mice that reached humane endpoints. Fixed tissue sections were stained with AFB plus H&E stain and evaluated by a veterinary pathologist (Figure 8, C–E). After MTBI, unvaccinated CC042 mice developed necrotizing inflammation with a paucity of lymphocytes and plasma cells, marked fibrin exudation in alveolar spaces, pyknotic nuclear debris (consistent with neutrophil and macrophage necrosis) that obstructed bronchioles, and massive necrosis (Figure 8C). Numerous extracellular AFB were primarily located extracellularly within inflammatory exudates, suggesting that MTBI induces inflammation that damages capillary endothelial cells and alveolar septa.

BCG vaccination of CC042 mice markedly changed the nature of the infiltrates. Vaccinated mice had fewer and smaller fibrinonecrotic foci surrounded by foamy macrophages and edema, and many prominent perivascular and peribronchiolar cuffs containing numerous lymphocytes and plasma cells (Figure 8D). AFB were detected in both intracellular and extracellular locations. Together with prolonged survival, these findings suggest that the main protective effects of BCG vaccination in the lungs are (a) reduced cellular necrosis and fibrin exudation; (b) increased numbers of lymphocytes, plasma cells, and macrophages; and (c) restriction of bacilli growth to intracellular compartments, likely by improved macrophage activation.

Histopathology from BCG-vaccinated, memory T cell–depleted mice was more variable. Mice that died early resembled unvaccinated CC042 mice, with fibrinonecrotizing inflammatory foci with necrotic neutrophils and/or macrophages occupying approximately 40%–60% of the lung area, with numerous extracellular AFB and few lymphocytes and plasma cells (Figure 8E). In contrast, the lung tissue from longer survivors was approximately 80% infiltrated with non-necrotic macrophages and foamy macrophages containing fewer, mostly intracellular, AFB and sparse lymphoid aggregates. These lungs did not contain much fibrin exudation or regions of alveolar septal necrosis.

To quantify lesion composition, we applied a machine learning model that was trained and validated using the Aiforia platform. Total tissue lung area, granuloma area, and percentage of tissue section occupied by granuloma were similar among groups (Supplemental Figure 9A). Regions of cell-poor necrosis and accumulation of pyknotic debris were ameliorated by BCG vaccination, irrespective of memory T cell depletion (Supplemental Figure 9B). BCG-vaccinated CC042 mice had approximately 4-fold more lymphocytes in lung lesions than unvaccinated mice. However, B6 mice had 10-fold more lymphocytes than BCG-vaccinated CC042 mice (Figure 8F). Lymphocyte cuff formation was stimulated by BCG vaccination and was associated with protection, but was independent of memory T cells. BCG-vaccinated mice had approximately 10-fold more lymphocytes in the cuff regions compared with unvaccinated mice, and this increase was abrogated when memory T cells were depleted before Mtb challenge (Figure 8F). Thus, the lymphocyte density in each cuff correlated with protection (~13-fold greater in vaccinated mice) and was dependent on anamnestic T cell responses (Figure 8F). Unexpectedly, plasma cells similarly correlated with protection and were T cell dependent (Figure 8H). Macrophages (Figure 8G) and neutrophils (Figure 8H) were significantly increased in the lesions of BCG-vaccinated mice, and their numbers were unaffected by memory T cell depletion.

Our model identifies 3 forms of Mtb: single bacterium, clusters, and biofilms. Single AFB and clusters were localized in macrophages or extracellular debris. Interestingly, the effect of vaccination led to a reduction in both extracellular AFB clusters and biofilms (Supplemental Figure 9, C and D) and an increase in intracellular single AFB (Supplemental Figure 9E). Thus, BCG vaccination of CC042 mice alters the long-term host response to Mtb that is observed at the time of death by increasing macrophage recruitment, reducing necrotic damage, and shifting the location of the Mtb bacilli intracellularly. These effects appear to be independent of conventional memory CD4^+^ and CD8^+^ T cells. Additionally, there is an increase in lymphocyte cuff formation and lymphocyte density, plasma cells, and neutrophils, which all appear dependent on memory T cells at the time of Mtb challenge.

## Discussion

The lack of CD11a expression increases the susceptibility of CC042 mice to MTBI (15). CD11a is crucial for lymphocyte trafficking and mediates cell extravasation across endothelium, immune synapse formation, and T cell activation (17, 32). Thus, pervasive lung and granuloma necrosis and early death following MTBI are consistent with CC042 mice having a defective T cell immunity (15). Surprisingly, CC042 is among several CC strains in which BCG vaccination led to a significant reduction in lung CFU 4 weeks after infection (10). We confirmed that subcutaneous BCG vaccination of CC042 mice leads to significant reductions in Mtb burdens in the lung, spleen, and lung-draining mLN in comparison with unvaccinated mice 4 weeks after low-dose aerosol MTBI, and increases their survival 3-fold. Since reduction in lung CFU and increased survival are standard parameters used to assess vaccine-induced protection in the murine TB model, we anticipated that studying CC042 mice would reveal non-canonical mechanisms of BCG-induced immunity.

We were surprised to observe increased lymphocytic infiltration in the lungs of BCG-vaccinated CC042 mice after Mtb challenge, with many of the lymphocytes being T cells based on flow cytometry. Protection persisted despite FTY720 treatment, raising the possibility that Trms populated the lung following vaccination, mediated by adhesion molecules other than LFA-1. However, depletion of CD4^+^ and CD8α^+^ T cells after vaccination, including antigen-experienced T cells in the lung, did not affect BCG’s ability to reduce Mtb burdens in CC042 mice. Similarly, depletion of B cells before vaccination did not affect protection. Although BCG-induced CFU reduction at 4 wpi is independent of CD4^+^ and CD8^+^ T cells and B cells, sustained protection beyond 4 weeks requires T cells. We show that the survival benefit conferred by BCG to CC042 mice is disrupted when BCG-induced T cells are depleted. Thus, we observe two distinct effector phases of immunity in CC042 mice: an initial phase that does not require the presence of CD4^+^ and CD8^+^ T cells, and a later T cell–dependent phase.

There remains considerable uncertainty regarding mechanisms of BCG-mediated protection against TB. Some theories posit that BCG primes adaptive immune cells for rapid and efficient recall responses to Mtb. BCG-primed T cells transfer protection to immunodeficient mice, although it is uncertain which subsets contribute to lasting immunity. While CD4^+^ T cells have long been correlated with protection, new approaches show a correlation between CD8^+^ T cells and immunity (33, 34). Other T cell subsets are generated during BCG vaccination, but their role in protection is incompletely understood (35, 36). Evidence from the C57BL/6 model generally argues against a role for B cells and antibodies in the mouse TB model; however, strong Th1 responses to mycobacteria may mask protective roles for B cells in this strain (37). Recently, evidence is accumulating that B cells may have a more important role in other mouse strains (38), humans, and nonhuman primates (NHPs) (39).

Other theories suggest that BCG enhances protection by trained innate immunity (31, 40). The concept arose from data showing that BCG vaccination at birth protects infants from pathogens other than Mtb, leading to a reduction in all-cause mortality in comparison with unvaccinated infants (30). The immune response to BCG leads to epigenetic modifications of HSCs, especially of the myeloid lineage (29, 41). BCG vaccination of B6 mice intravenously, but not subcutaneously or intranasally, induces “training,” and BM-derived macrophages have an enhanced ability to restrict Mtb growth in vitro (31). The intravenous route requirement appears to reflect a need for BCG to enter the BM compartment. Innate immune training is IFN-γ dependent, and could require T cells, although other sources of IFN-γ exist (42). So, while BCG can induce innate immune training, it is not yet clear whether BCG consistently disseminates to the BM in mice, NHPs, or humans following subcutaneous vaccination. Besides epigenetic modification of HSCs, BCG can also modify alveolar macrophage responses (43). It is not yet clear whether innate training leads to durable protection or whether it could be a major mechanism of protection in vivo.

Although animal models do not fully recapitulate human disease, they are indispensable for vaccine development, because they allow for standardized testing of vaccine efficacy and mechanisms. Standardization efforts led to widespread use of a consistent Mtb challenge strain (low-dose aerosolized Mtb Erdman) and analysis at 30 days after infection (44). Increasingly, investigators question how to interpret early time points, as BCG-induced protection is often transient. Similarly, the dominant use of C57BL/6 and BALB/c mice in vaccine studies risks optimizing immunization strategies for specific genetic backgrounds. An ongoing debate is whether to use susceptible mouse strains (45, 46). Previously, there was resistance to the use of susceptible strains such as C3H substrains because of concern that they had defective immunity; however, their development of necrotic and hypoxic lung lesions is more like human granulomas (47, 48).

Surprisingly, despite the absence of CD11a, CC042 mice generate complex immune responses to BCG, which permits evaluation of several disease and immunological parameters following BCG vaccination and Mtb challenge. Protection induced by BCG vaccination of CC042 mice is based on 3 parameters: reduced bacillary load; better Mtb containment and less lung disease; and increased survival. A future question is whether these different parameters are interdependent or reflect different disease processes and mechanisms of protection. Finding that CD4^+^ and CD8^+^ T cells are dispensable for BCG-induced protection 4 weeks after Mtb challenge is surprising since this is a standard time point to assess vaccine-induced protection in the mouse TB model (48). As we are depleting T cells after BCG vaccination but before Mtb challenge to provide time for reconstitution of the T cell compartment, we are primarily removing memory T cells from the immune repertoire and preventing T cell recall responses (i.e., secondary responses) from developing following Mtb challenge. This approach was validated by our demonstration that the recall response to MTBI was abrogated in BCG-vaccinated CC042 mice that had been treated with anti-CD4/8 (Figure 4E). Consequently, we infer that this early phase of protection is primarily mediated by non–T cell mechanisms of immunity. Although CD4^+^ and CD8^+^ T cells are not acting as effectors during this phase of infection, it appears that T cells are orchestrating protective immune responses. It is possible that 12 weeks of anti-CD4/8 mAb treatment led to more profound T cell depletion than the 3-week course, or caused off-target effects. However, profound depletion of T cells prior to infection was evident after both the 3-week and the 12-week treatment course. Furthermore, the CFU in untreated (No Tx) versus T cell depleted and treated with antibiotics (T-depletion + INH/Rif) (Figure 7E) were similar, which suggests that the treatment did not degrade control of MTBI by CC042 mice. Depletion of CD4^+^ and CD8^+^ T cells before vaccination prevented the development of protective immunity, which implies that T cells enhance innate responses to control Mtb early during infection (i.e., 4 wpi). In contrast, long-term protection (i.e., survival) requires T cells. In our 4-week mechanistic studies, CC042 mice were challenged with H37Rv.YFP to facilitate determination of the distribution of Mtb among myeloid cell types 4 weeks after infection, and to compare these results with those of our prior experiments with CC mice (10, 14). In contrast, CC042 mice in the survival study were infected with Mtb Erdman, as H37Rv.YFP is less useful for long-term infections. Since H37Rv.YFP is less virulent than Mtb Erdman, one question is whether T cells have a greater role in protection against virulent Mtb strains. A focus of many vaccine studies is the identification of immune correlates of protection. Reliance on a single early time point would have limited our analysis and failed to uncover a role for T cells in BCG-mediated protection of CC042 mice.

While we have not formally identified the immune mechanisms that operate during the initial 4 weeks, they are likely to be innate or innate-like immune mechanisms (e.g., innate training, NK cells, or donor-unrestricted T cell responses). We focused on immunity mediated by conventional CD4^+^ and CD8^+^ T cells (i.e., class II and class I MHC restricted, respectively) as there is agreement that these T cells are essential for immunity to Mtb (49). Other T cells lack CD4 or CD8 expression and would not be directly affected by CD4/CD8α antibody depletion. These include MR1-restricted T cells (MAIT cells) and CD1d-restricted T cells (NKT cells), which are sometimes referred to as donor-unrestricted T cells (DURTs) (50) because their restricting elements (e.g., MR1, CD1d) are non-polymorphic compared with MHC. The majority of γδ T cells also lack CD4 and CD8 expression, and although the understanding of how γδ T cells recognize Mtb antigens is incomplete, γδ T cell expansions are frequently associated with TB (51). In humans, substantial γδ T cell responses, but not DURT responses, are detected after BCG vaccination (35, 52, 53). There are few data on how BCG affects these T cell subsets in experimentally infected animals, and there are important species-specific differences (54). As a new murine resource, relatively little is known about DURTs and γδ T cells in CC strains, although one strain, CC011 mice, has an expanded population of MAIT cells (55). Whether non-conventional T cells play a role in BCG-induced protection against Mtb is an important question for future investigation. While most data support a requirement for LFA-1 in the function of MAIT, NKT, and γδ T cells (56–59), we cannot yet exclude the possibility that these T cells contribute to BCG-mediated protection by an LFA-1–independent mechanism in CC042 mice.

CC042 mice are a complex mosaic of 8 highly diverse parental founders. While a defective *Itgal* gene is an important driver of their susceptibility to Mtb and *Salmonella* (15, 60), the 15-base intronic deletion is a private mutation that is present in CC042 mice but not any of the founders. There are other genetic loci that affect the susceptibility of CC042 mice to MTBI other than the *Itgal* gene. Indeed, CC042 mice are more susceptible than CD11a^–/–^ mice (backcrossed to B6 mice), and the nature of their lung lesions differs considerably (19). Nevertheless, these data show that CD11a is not necessary for BCG-mediated protection against MTBI, and immune mechanisms other than conventional B and T cells can be sufficient to control MTBI. Based on these studies, we expect that interrogating how genetic diversity affects immunity should provide insight into host resistance against MTBI.

## Methods

### Sex as a biological variable.

Susceptibility of CC042 mice to MTBI and BCG-mediated protection was not affected by sex (see Figure 1). Therefore, sex was not analyzed as a variable in this study, and mice of both sexes were used in the experiments and had similar results.

### Mice.

Sex- and age-matched mice, born within 3 weeks of each other, were used for experiments. Six- to eight-week-old B6 mice were purchased from The Jackson Laboratory. CC042 (CC042/GeniUnc) mice were obtained from the University of North Carolina Systems Genetics Core Facility (Chapel Hill, North Carolina, USA) and bred locally. All animal studies were conducted in the Animal Facility at the University of Massachusetts Chan Medical School.

### In vivo infections.

Frozen Mtb stocks of Rv.YFP (H37Rv expressing yellow fluorescent protein sfYFP) (61) or Erdman were thawed, sonicated for 1 minute, diluted into 5 mL of CFU buffer (0.01% Tween 80, Thermo Fisher Scientific) in PBS (Gibco), and administered by aerosol generated by the Glas-Col Inhalation Exposure system. Lung CFU assays (*n* = 4–5) performed within 24 hours after infection confirmed the average bacterial inoculum, which ranged from 20 to 119 (Mtb Rv.YFP) and 17 to 100 (Erdman) CFU per mouse.

### BCG vaccination.

Frozen stocks of BCG-SSI were thawed on ice for 30 minutes and then triturated through a 22-gauge needle (BD Biosciences) on a 1 mL syringe (BD Biosciences) 10 times to disrupt clumps. The bacterial suspension was diluted in PBS to administer 500,000 CFU of BCG in 100 μL (flank) or in 20 μL (hock).

### Survival analysis.

Mice were weighed and monitored at least once weekly using the body condition score (BSC). Mice with a BSC less than or equal to 2 or weight less than 80% baseline were euthanized.

### In vivo depletions.

For CD4^+^ and CD8α^+^ cells, mice received both anti-CD4 (GK1.5) and anti-CD8α (2.4.3) mAbs (Bio X Cell) intraperitoneally twice weekly during the depletion period. The first dose contained 200 μg of each mAb suspended in 200 μL of PBS, and all subsequent doses contained 100 μg of each mAb suspended in 200 μL of PBS.

For CD20^+^ cells, mice received 250 μg of anti-CD20 mAb (MB20-11, Bio X Cell) suspended in 200 μL of PBS intraperitoneally 1 week before vaccination and again 6 weeks later.

For cross-blocking analysis, splenocytes from CC042 mice were individually incubated with 100 μg of each unlabeled antibody (anti-CD20, MB20-11; anti-CD4, GK1.5; or anti-CD8α, 2.4.3) for 20 minutes at room temperature. Cells were then washed once with PBS, stained with fluorescently labeled mAbs specific for CD20, CD4, or CD8, as indicated in Supplemental Figure 4A, and then analyzed by flow cytometry.

### Bacterial burden determination.

Vaccinated and infected mice were euthanized at the indicated time points. The left lung lobe, mediastinal LN, inguinal LN, or spleen was homogenized with 2 mm zirconium oxide beads (Next Advance) in a FastPrep homogenizer (MP Biomedical), 3 rounds for 20 seconds each. Homogenates were then serially diluted and plated on 7H11 plates (Hardy Diagnostics). CFU were enumerated 21–25 days after incubation at 37°C.

### Cell preparation.

Lung single-cell suspensions were prepared first by homogenizing in a GentleMACS tissue dissociator (Miltenyi Biotec) and incubated in collagenase (300 U/mL; MilliporeSigma) in 6 mL of total RPMI (Gibco) for 30 minutes. Lungs were homogenized again in the GentleMACS, then sequentially filtered through 70 μm and 40 μm cell strainers (Thermo Fisher Scientific) and suspended in complete RPMI (cRPMI) with 10% FBS (Gibco), 2 mM l-glutamine (Gibco), 50,000 units penicillin/streptomycin (Gibco), 1 mM sodium pyruvate (Gibco), 1× non-essential amino acids (Gibco), 1× essential amino acids (Gibco), 25 mM of HEPES (Gibco), 7.5 mM of sodium hydroxide, and 0.55 μM 2-mercaptoethanol (Gibco). Spleen and LN single-cell suspensions were prepared by manual homogenization with plungers from 5 mL syringes (Thermo Fisher Scientific), sequentially filtered through 70 μm and 40 μm cell strainers, and suspended in cRPMI. Lung and spleen cells were treated with ACK lysis buffer (Gibco) for 1–2 minutes before filtering through 40 μm strainers. BM cells were acquired by flushing of tibiae and femora with PBS, then filtering of the cells through a 70 μm cell strainer. Cells from single-cell suspensions were counted on a TC20 (Bio-Rad) at 1:20 dilution in PBS.

### Blood lymphocyte preparation.

Blood was collected via cheek bleeds in 2 mL of RPMI containing 80 units of heparin (Thermo Fisher Scientific). The blood was underlain with 1 mL of Lympholyte (Cedarlane) and centrifuged for 20 minutes at 400 X g. The lymphocyte layer was transferred to a fresh tube containing 2 mL of autoMACS (Miltenyi Biotec) running buffer and washed once with PBS before being stained with antibodies.

### Serum collection.

Blood was collected from the vena cava at sacrifice, allowed to clot overnight at 4°C, and centrifuged at 2,000*g* for 10 minutes at 4°C. The serum was collected, filtered through a Multiscreen 96-well 0.22 μm filter plate (Millipore), and stored at –80°C.

### Antibody ELISA.

Polystyrene 96-well plates were coated with 10 μg WCL or CFP (BEI Resources) in PBS overnight at 4°C. Plates were blocked with 100 μL per well of 1% bovine serum albumin (BSA; Thermo Fisher Scientific) in PBS plus 50 μL of serum diluted 1:40 in 1% BSA, followed by adding 50 μL of alkaline phosphatase–conjugated goat anti-mouse IgG (H+L) (Jackson ImmunoResearch Laboratories; diluted 1:1,000 in 1% BSA) to each well and then 50 μL of phosphatase substrate (MilliporeSigma) for up to 30 minutes. The reaction was quenched with 1 M sodium hydroxide (Spectrum Chemical) in water, and absorbance was measured at OD_405_. All incubations were performed at room temperature with gentle shaking, and plates were washed 3 times with wash buffer (PBS plus 0.1% Tween 20 [Thermo Fisher Scientific]) between steps.

### ELISPOT.

All steps were followed according to the manufacturer’s protocol for the Mouse IFN-γ ELISPOT Set from BD Biosciences. Briefly, 96-well ELISPOT plates were coated overnight at 4°C with purified IFN-γ in dilution buffer (PBS with 10% FBS) and blocked with blocking solution (RPMI supplemented with 10% FBS, 50,000 units of penicillin/streptomycin, and 2 mM l-glutamine) for 2 hours at room temperature. Two hundred thousand popliteal lymph node cells were seeded per well of the coated plate and cocultured with 2 μg/mL of P300 mycobacterial peptide pool or 10 μg/mL of Ag85B_241–256_ peptide in cRPMI. The cells were incubated for 44 hours at 37°C with 5% CO_2_. After incubation, cell suspensions were aspirated, and the plate was incubated with diluted detection antibody in dilution buffer for 2 hours at room temperature. The plate was incubated for 1 hour at room temperature with enzyme conjugate diluted in dilution buffer and then incubated with the substrate solution diluted in dilution buffer until spots formed. The substrate reaction was quenched with deionized water and allowed to dry, and spots were enumerated on the S5 CTL ImmunoSpot. All incubations at room temperature were performed with gentle shaking, and plates were washed 3 times with wash buffer or PBS between steps.

### Flow cytometry.

Cells were washed once with PBS, stained with Zombie viability dye (BioLegend) for 10 minutes at room temperature, washed with autoMACS running buffer, and incubated with 1% anti–mouse CD16/32 (BioLegend) suspended in autoMACS running buffer for 10 minutes at room temperature. When applicable, cells were washed again with autoMACS running buffer, then incubated with MR1 and CD1d tetramers suspended in cRPMI for 1 hour at 37°C. Surface staining was performed by washing of cells with autoMACS running buffer, incubation of cells with antibodies (Supplemental Table 1) suspended in autoMACS running buffer for 20 minutes at 4°C, and fixation of cells in 1% paraformaldehyde (Thermo Fisher Scientific) suspended in PBS for at least 20 minutes. Samples were acquired on the Miltenyi Biotec MACSQuant 16 or the Cytek Aurora. All data were analyzed with FlowJo software, version 10 (Waters Bioscience).

### Intravascular staining.

None of the available anti-CD45 mAbs stained CC042 leukocytes. Therefore, to distinguish intracellular versus parenchymal leukocytes in the lungs of Mtb-infected CC042 mice, leukocytes were injected intravenously with 1.5 μg/mouse each of CD90.2, CD19, and NKp46 (AF647, BioLegend) suspended in 200 μL total volume of 2% FBS in PBS. Mice were euthanized exactly 3 minutes after injection, and lungs were harvested.

### Antibiotic treatment.

Mice received drinking water containing 0.5 g/L of isoniazid (MilliporeSigma, from 250× stock in water) and 0.1 g/L of rifampicin (MilliporeSigma, from 250× stock in DMSO [Thermo Fisher Scientific]) during the specified time periods. Medicated water was protected from light and replaced weekly.

### Histopathological tissue preparation.

Lungs were inflated and immersion-fixed immediately after euthanasia with Z-fix (Anatech) for at least 30 minutes and then washed with PBS. Tissue was embedded in paraffin, sectioned, affixed to glass slides, and dewaxed in an oven at the Morphology Core Facility at University of Massachusetts Chan Medical School. Fixed tissue was deparaffinized in 1 part peanut oil (Planters) to 2 parts xylene (Thermo Fisher Scientific) twice for 12 minutes each. Tissue was stained with carbol fuchsin (5% phenol, 10% ethanol, 1% basic fuchsin in distilled water) for 25 minutes, differentiated in acid alcohol (1% hydrochloric acid in 70% ethanol), stained with Harris hematoxylin with glacial acetic acid (Polysciences) for 3 minutes, neutralized in bluing reagent (0.25% ammonium hydroxide in distilled water), and counterstained with eosin phloxine alcoholic working solution (Polysciences). Slides were dehydrated in ethanol (Fisher Scientific), then xylene, and coverslipped with Permount (Fisher Scientific).

### Histopathological tissue evaluation.

Stained tissue sections were digitally scanned by Aperio ScanScope or AT2 scanners at 0.23 μm/pixel at Vanderbilt University Medical Center’s Digital Histology Shared Resource (Nashville, Tennessee, USA). Digital images were uploaded to the Aiforia Create (v6.0) platform (Aiforia Technologies) Slide Viewer for blinded qualitative evaluation and semiquantitative assessment of tissue architecture, cell types, and AFB by a board-certified veterinary pathologist.

### Automated image analysis of histopathological tissue.

Manual training annotations were provided using the Aiforia Create (v6.0) platform with default parameter settings to create a multilayer, multiclass convolutional neural network–based model for automated image analysis to detect and quantify lung tissue, granulomas, subregions of granulomas, i.e., pyknotic debris, cell-poor necrosis, lymphocytic cuffs, fibrin, and viable regions of granulomas containing foci of immune cells and their nuclei; and AFB single bacilli, clusters of bacilli, and regions where bacilli formed a mat resembling a biofilm containing organisms too numerous to count. Many iterative rounds of training, testing, and validation by pathologists’ inspection of model performance on independent images (not used in training) were performed until errors were minimized.

### Statistics.

Statistical analyses used GraphPad Prism (v10). *P* values were calculated using unpaired or paired 2-tailed *t* test, 1-way ANOVA, or 2-way ANOVA with post hoc tests as indicated in the figure legends. A *P* value of less than 0.05 was considered significant.

### Study approval.

Studies were approved by the Institutional Animal Care and Use Committee at the University of Massachusetts Chan Medical School (Animal Welfare A3306-01), using the recommendations from the *Guide for the Care and Use of Laboratory Animals* (National Academies Press, 2011) of the NIH and the Office of Laboratory Animal Welfare.

### Data availability.

A Supporting Data Values file is available as supplemental material. Any additional information required to reanalyze the data reported in this paper is available upon request. Requests for further information and for resources and reagents should be directed to and will be fulfilled by the corresponding author.

## Author contributions

AFO and SMB designed research. AFO, AVT, RL, and KC performed research. AFO, GLB, and SMB analyzed data. AFO and SMB wrote the manuscript. All authors reviewed and edited the manuscript. SMB acquired funding for the research.

## Conflict of interest

The authors have declared that no conflict of interest exists.

## Funding support

This work is the result of NIH funding, in whole or in part, and is subject to the NIH Public Access Policy. Through acceptance of this federal funding, the NIH has been given a right to make the work publicly available in PubMed Central.

- NIH/National Institute of Allergy and Infectious Diseases grants R01 AI123286 (to SMB), R01 AI172905 (to SMB and GLB), R01 HL145411 (to GLB), and contract 75N93019C00071 (to SMB).
- NIH Immunology and Microbiology Training Grant T32AI007349 (to AFO).
- NIH Medical Scientist Training Program Grant T32GM159591 (to AFO).

## Supplementary Material

Supplemental data

Supporting data values

## Version 1 07/02/2026

In-Press Preview

## Version 2 08/24/2026

Electronic publication
