# Supplementary material for: BCG vaccination elicits protection against M. tuberculosis infection mediated by two phases of T cell immunity
Source: JCI Insight. 2026 Jul 2;11(16):e201000. doi: 10.1172/jci.insight.201000 (PMC13505383; doi:10.1172/jci.insight.201000)
Supplement: Supplementary file 1 [file jciinsight-11-201000-s014.pdf]

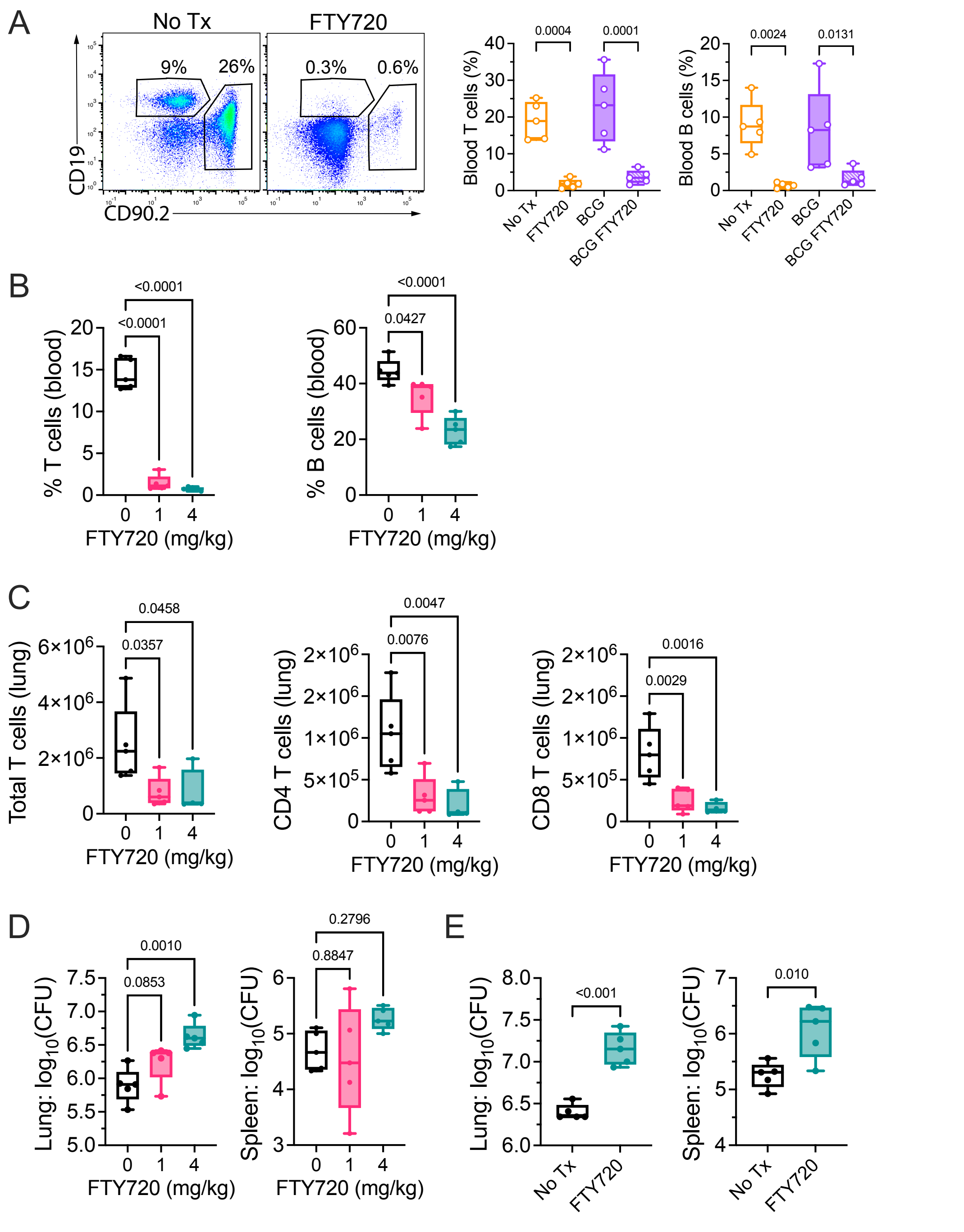
A
No Tx
FTY720
9%
26%
0.3%
0.6%
CD19
CD90.2
Blood T cells (%)
0.0004
0.0001
No Tx
FTY720
BCG
BCG FTY720
Blood B cells (%)
0.0024
0.0131
B
% T cells (blood)
<0.0001
<0.0001
FTY720 (mg/kg)
% B cells (blood)
<0.0001
0.0427
C
Total T cells (lung)
0.0458
0.0357
CD4 T cells (lung)
0.0047
0.0076
CD8 T cells (lung)
0.0016
0.0029
D
Lung: log10(CFU)
0.0010
0.0853
Spleen: log10(CFU)
0.2796
0.8847
E
Lung: log10(CFU)
<0.001
Spleen: log10(CFU)
0.010

Figure S1

Figure S1. Optimization of FTY720 use in the murine TB model (pertains to Fig.3)

A. Treatment of B6 mice with FTY720 after Mtb infection led to a reduction in the percentage of T cells (left) and B cells (right) among all live cells in peripheral blood as determined by flow cytometry.

B. Treatment with FTY720 led to significant reductions in the frequency of both T (left) and B (right) cells in the blood of B6 mice after Mtb infection.

C. Treatment with FTY720 led to significant reductions in the number of total T cells (left) including CD4 (middle) and CD8 (right) T cells in the lungs of Mtb infected B6 mice.

D. The bacterial burden in the lungs (left) or spleens (right) of infected B6 mice was increased when treated with 4 mg/kg of FTY720, but not 1 mg/kg of FTY720, as determined four wpi. FTY720 did not have a significant effect on Mtb control in the spleens of treated mice. One-way ANOVA with Dunnett's multiple comparisons test.

E. CFU in the lung (left) and spleen (right) of B6 mice at four wpi. This experiment performed in parallel with the one with CC042 mice depicted in Figure 3.

n=4-5 mice/group. One-way ANOVA with Dunnett's multiple comparisons test (A-D) or t-test (E). Data is representative of one (A-D) or two (E) individual experiments. Box plots indicate median (middle line), 25th, 75th percentile (box) and minimum and maximum (whiskers).

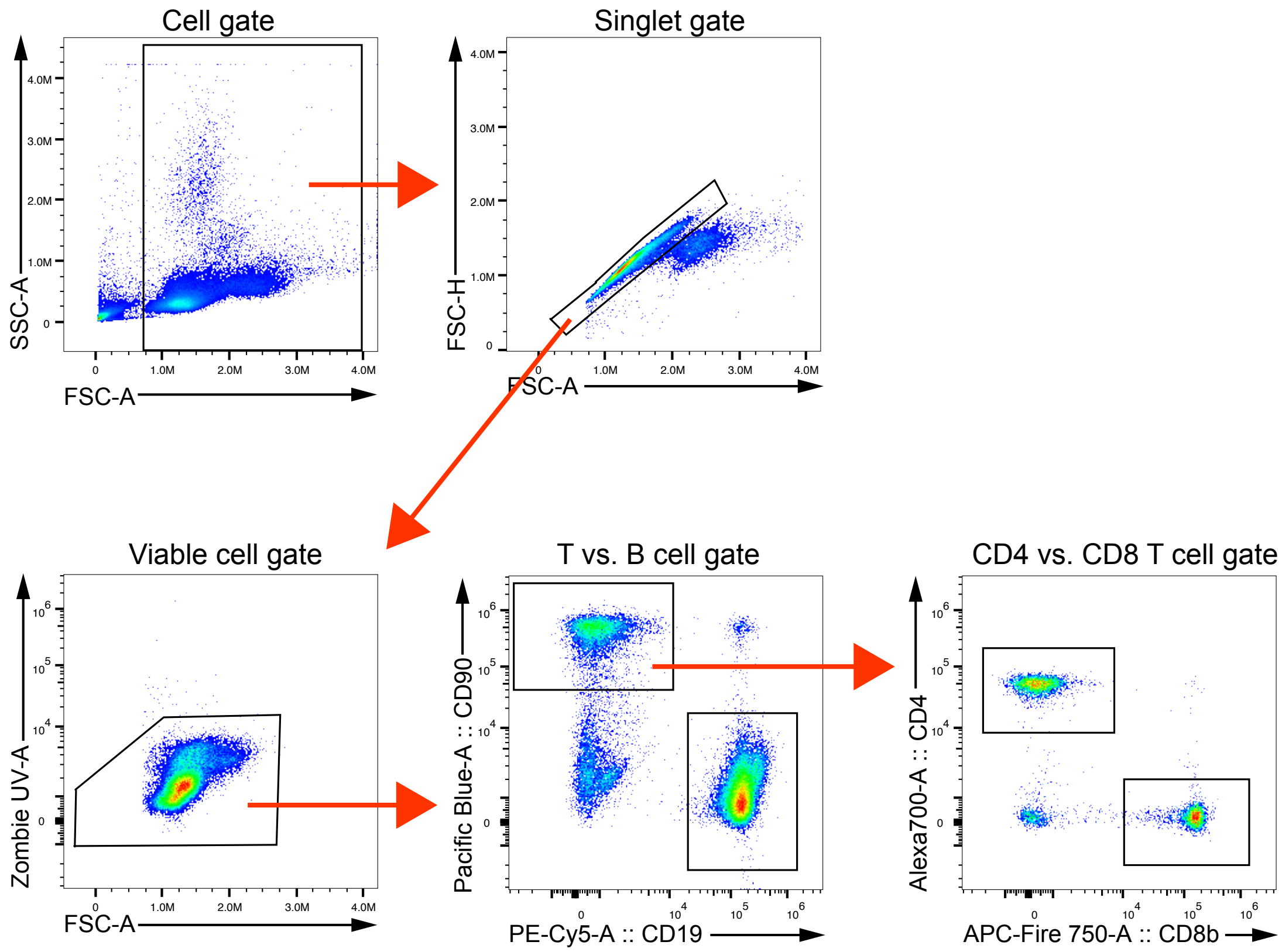

Figure S2. Gating Scheme for Identifying B & T cells in Peripheral Blood

Gating strategy for identifying B and T cells in the peripheral blood. In brief, doublets were excluded and then a viability dye was used to exclude dead cells. B cells were identified by expression of CD19 while T cells were identified by expression of CD90 or CD3. Then, CD4 and CD8α or CD8β was used to specifically identify CD4 T and CD8 T Cells.

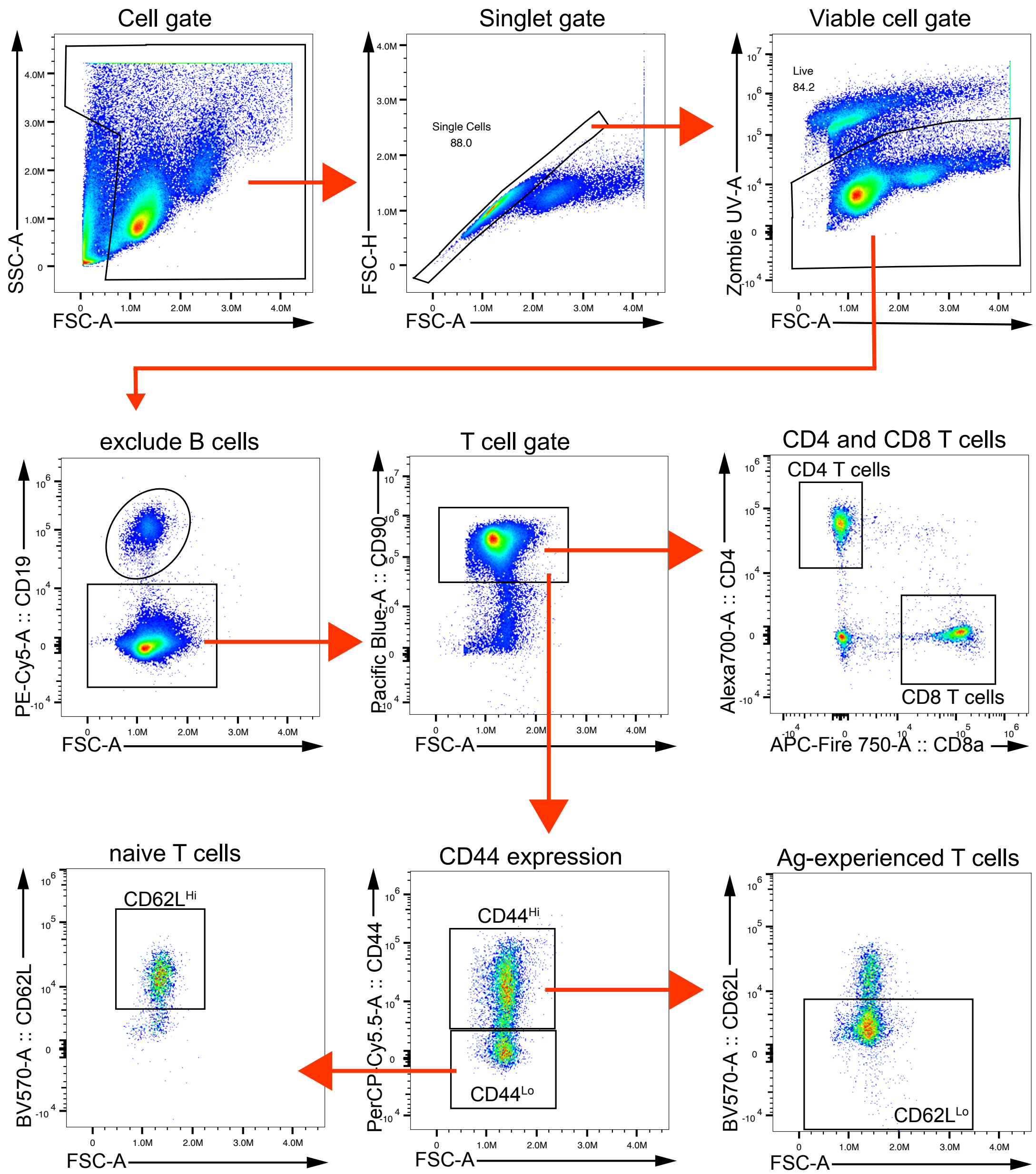

Figure S3. Gating Scheme for Identifying T cells in Lung

Gating strategy for identifying T cell population in the lung. In brief, doublets were excluded and then a viability dye was used to exclude dead cells. B cells were then excluded by expression of CD19, while T cells were identified by expression of CD90. T cells were then subclassified by CD4 or CD8 by their expression of CD4 or CD8α or CD8β, respectively. Naïve T cells were identified by the expression of CD62L and lack of CD44 expression. Antigen-experienced T cells were identified by expression of CD44 and lack of expression of CD62L.

A

Specificity	Primary antibody (in vivo depleting)	Secondary antibody (in vitro staining)	Cross-blocking?
CD4	GK1.5	GK1.5<br>RM4-4<br>RM4-5	yes<br>no<br>no
CD8a	2.4.3	53-6.7<br>YTS156.7.7	no<br>no
CD20	MB20-11	6D5	no

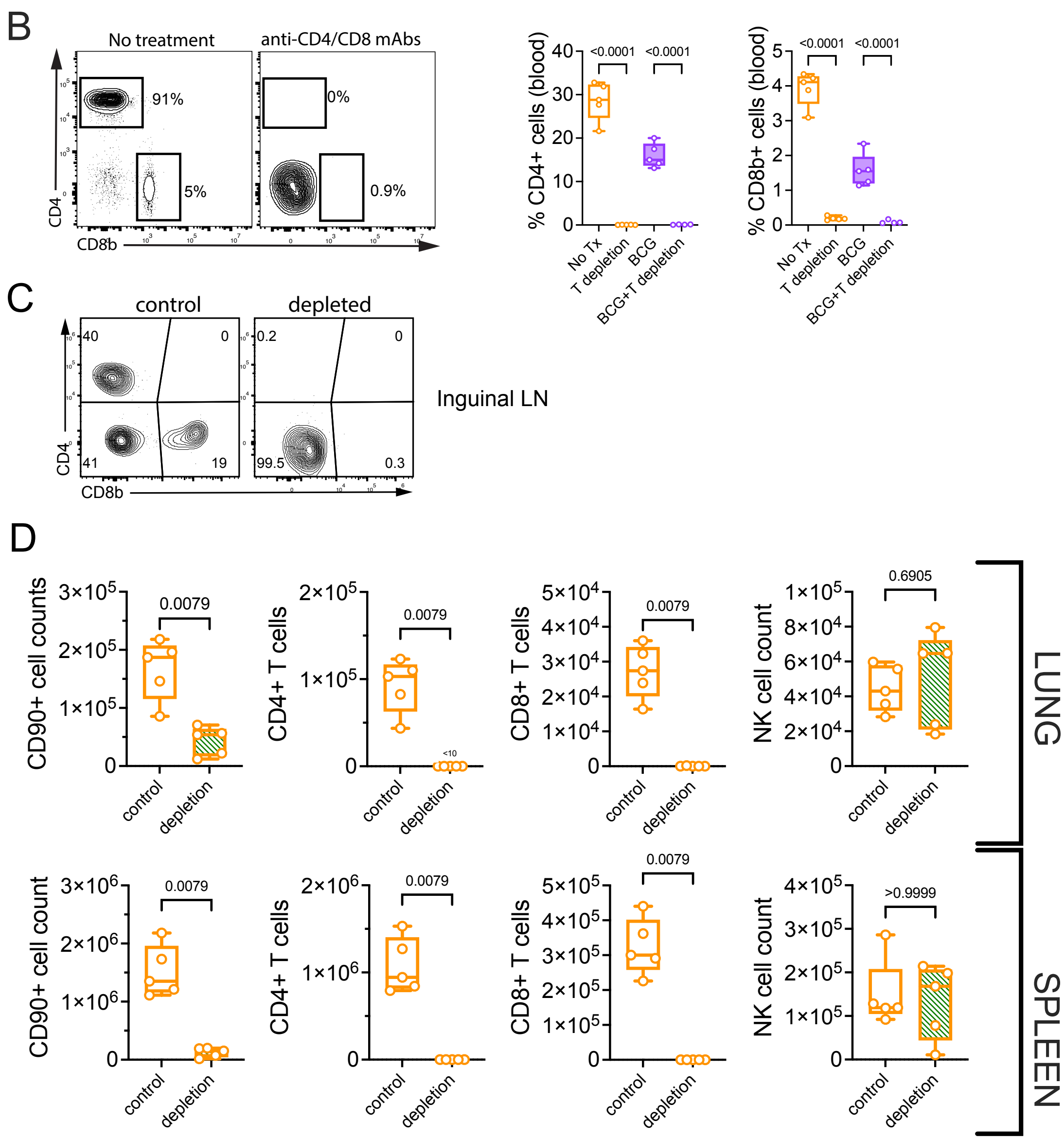

Figure S4

Figure S4. T cell depletion strategies (pertains to Fig.4)

A. Evaluation of antibody cross-blocking potential in CC042 mice. The anti-CD4 mAb GK1.5 that is used to deplete CD4 T cells in vivo blocked the binding of labeled GK1.5, but not of alternate anti-CD4 mAbs RM4-4 or RM4-5. The anti-CD8α mAb used in vivo (clone 2.4.3) does not block binding of alternate anti-CD8α clone 53-6.7 or anti-CD8β clone YTS156.7.7. Therefore, the efficiency of CD4 and CD8 T cell depletion in vivo by GK1.5 and 2.4.3 was quantified using anti-CD4 mAbs RM4-4 or RM4-5 and anti-CD8α mAb 53-6.7 or CD8β YTS156.7.7 in all T cell depletion experiments. Similarly, the anti-CD20 mAb MB20-11, used to deplete B cell in vivo, did not inhibit the binding of anti-CD19 mAb clone 6D5 to B cells.

B. Representative flow plot of CD4 and CD8β expression of $CD90^+$ T cells in the blood at four wpi of a representative untreated CC042 mouse and one treated with anti-CD4 and anti-CD8α mAbs (left). Frequency of $CD90^+$ CD4 and CD8β T cells in blood at the end of the depletion period before Mtb infection of CC042 mice (Right).

C. Representative flow plot demonstrating CD4 and CD8 T cell depletion in the inguinal LN from BCG-vaccinated CC042 mice after treatment with anti-CD4 and anti-CD8 mAbs as described in the *Methods.*

D. Depletion of CD4 and CD8 T cells from the lungs (top) and spleens (bottom) of CC042 mice. CC042 mice were vaccinated with BCG and treated with anti-CD4 and anti-CD8 mAbs as described in the *Methods.* Cells were stained with markers for CD4 and CD8 T cells, and NK (NKp46) cells.

One-way ANOVA with Šídák's multiple comparisons test (B) Or Mann-Whitney t-test (D). Data are representative or two (D) or three (B, C) independent experiments with similar results. Each point represents an individual subject, n=5 mice per group. Box plots indicate median (middle line), 25th, 75th percentile (box) and minimum and maximum (whiskers).

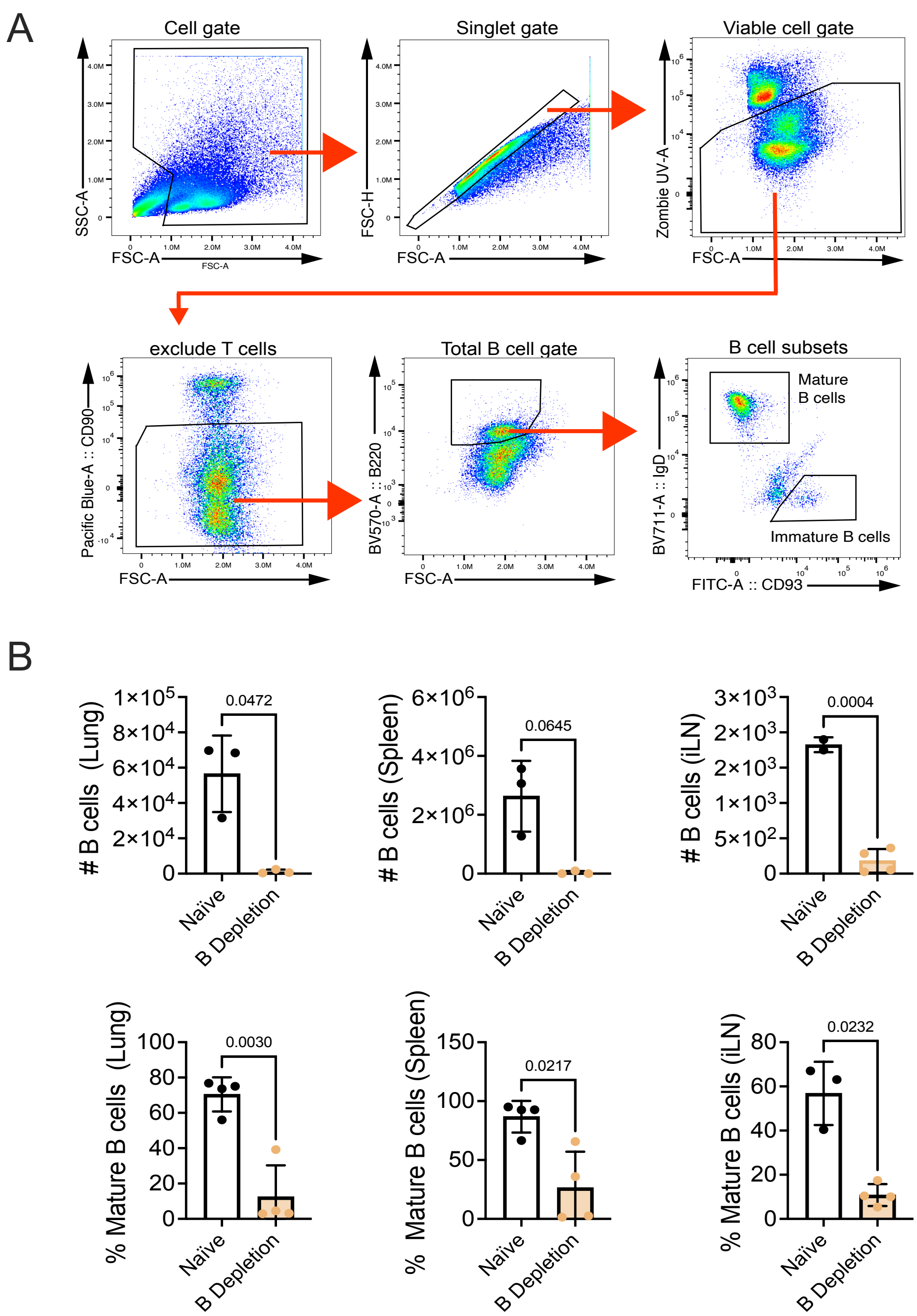
A
Cell gate
Singlet gate
Viable cell gate
SSC-A
FSC-A
FSC-H
Zombie UV-A
exclude T cells
Total B cell gate
B cell subsets
Pacific Blue-A :: CD90
BV570-A :: B220
BV711-A :: IgD
FITC-A :: CD93
Mature
B cells
Immature B cells
B
B cells (Lung)
B cells (Spleen)
B cells (iLN)
% Mature B cells (Lung)
% Mature B cells (Spleen)
% Mature B cells (iLN)
0.0472
0.0645
0.0004
0.0030
0.0217
0.0232
Naïve
B Depletion

Figure S5

Figure S5. B cell depletion strategies (pertains to Fig.5)

A. Gating strategy for identifying B cell populations in the lung, lymph node and spleen. In brief, doublets were excluded and then a viability dye was used to exclude dead cells. T cells were excluded by the expression of CD90. Total B cells were identified by the expression of B220. B cells were then further classified as mature or immature. Mature B cells were identified by expression of IgD and lack of CD93 expression, while immature B cells were identified by expression of CD93 and lack of IgD expression.

B. Number of total B cells (top row) and frequency of mature ($B220^{+}IgD^{+}CD93^{-}$) B cells as a percentage of total B cells (bottom row) in the lung (left), spleen (middle) and iLN (right), two weeks after a single administration of anti-CD20 mAb to naïve CC042 mice. Data represents two experiments. Each point is an individual subject, n=3-4 mice per group. Unpaired t-test. Bars, mean. Line, SD.

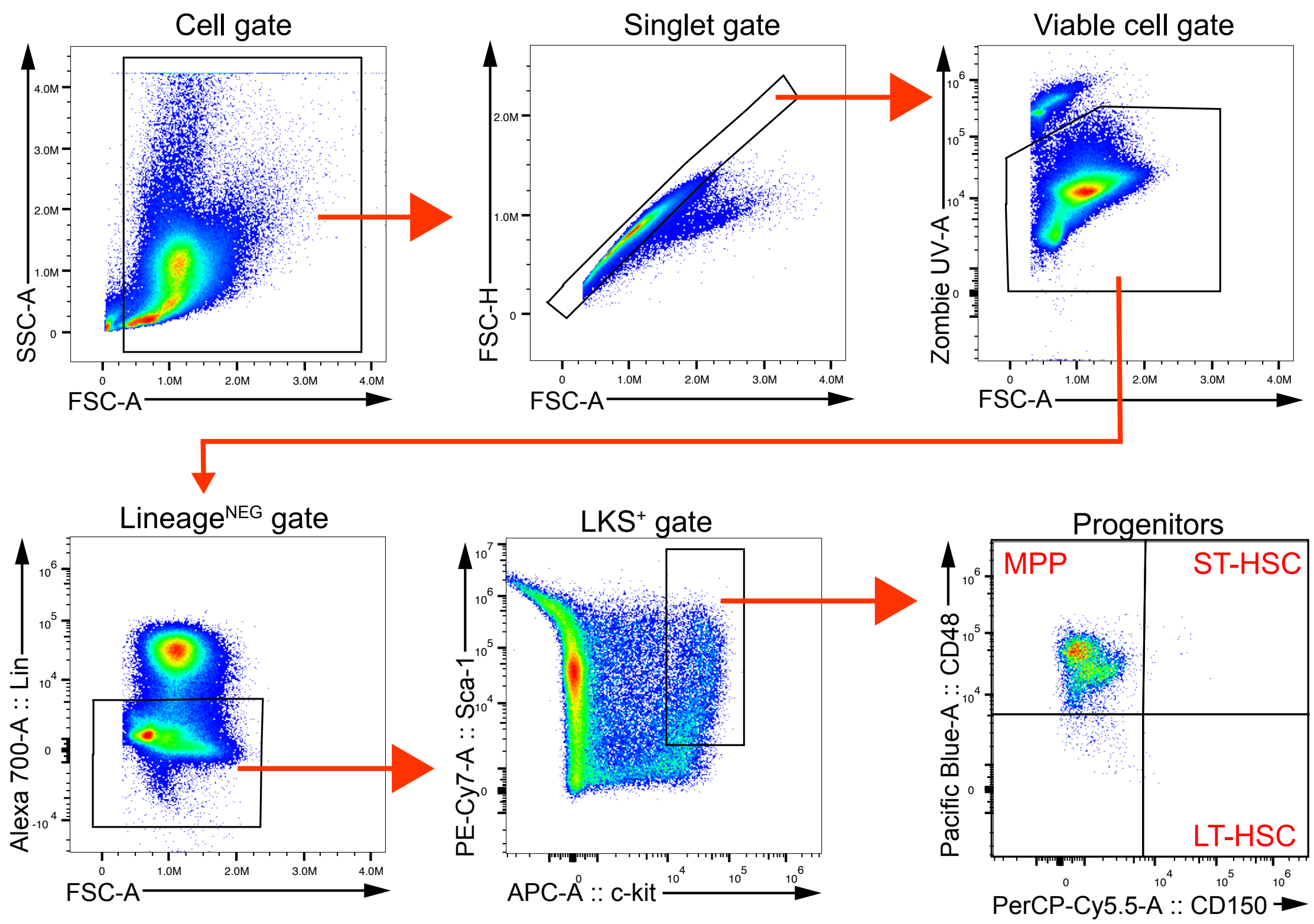

Figure S6. Gating Scheme for Identifying LKS+ Cells in Bone Marrow

Gating strategy for identifying LKS+ hematopoietic stem cells in the bone marrow. Doublets were excluded and then a viability dye was used to exclude dead cells. LKS+ cells were then identified by the lack of expression of a lineage marker and co-expression of Sca-1 and c-kit. LKS+ cells were then further sub-divided into MPP, ST-HSC and LT-HSC by differential expression of CD48 and CD150.

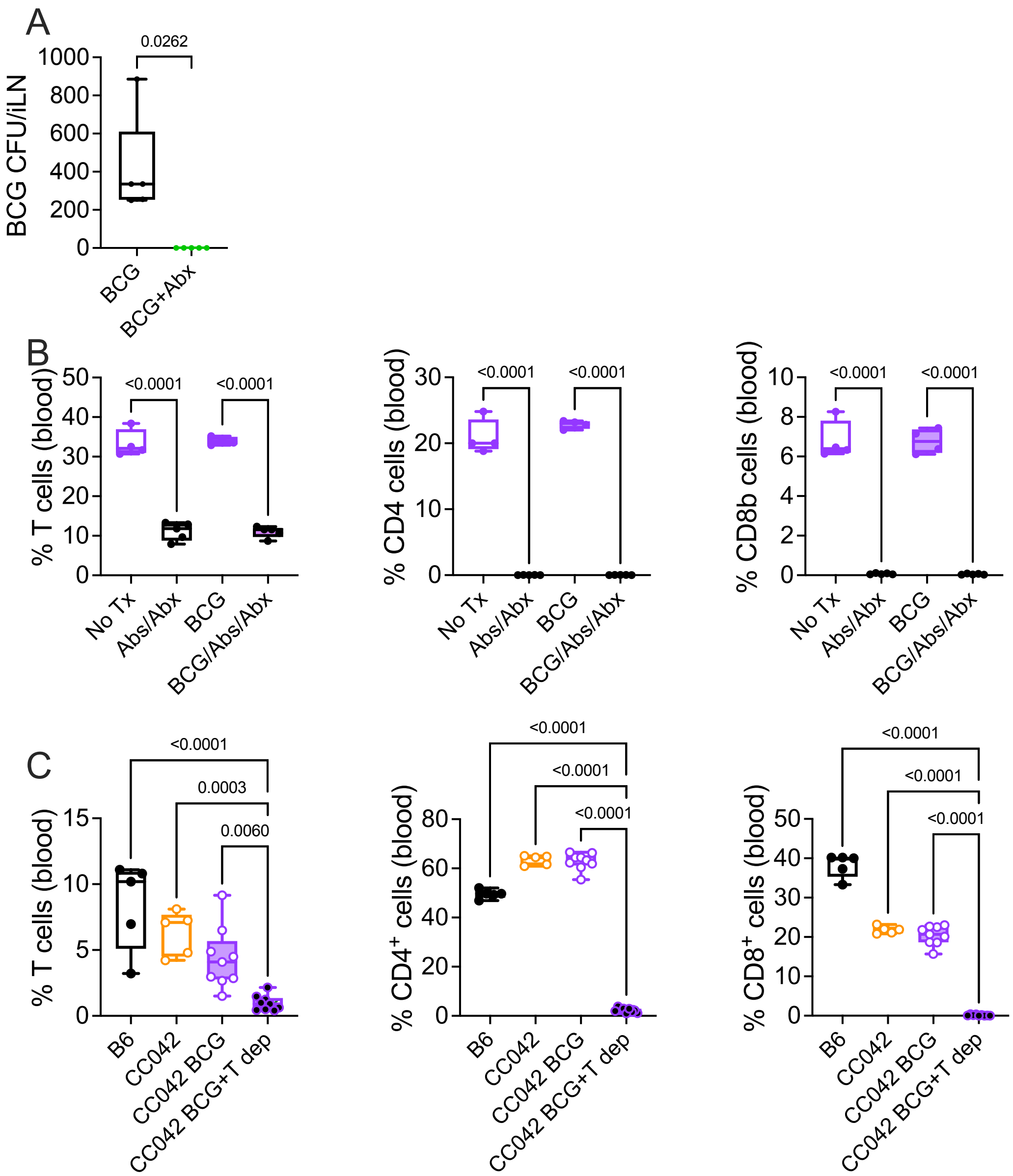
A
0.0262
BCG CFU/iLN
1000
800
600
400
200
0
BCG
BCG+Abx
B
% T cells (blood)
50
40
30
20
10
0
<0.0001
<0.0001
No Tx
Abs/Abx
BCG
BCG/Abs/Abx
% CD4 cells (blood)
30
20
10
0
% CD8b cells (blood)
10
8
6
4
2
0
C
% T cells (blood)
15
10
5
0
<0.0001
0.0003
0.0060
B6
CC042
CC042 BCG
CC042 BCG+T dep
% CD4+ cells (blood)
80
60
40
20
0
<0.0001
% CD8+ cells (blood)
50
40
30
20
10
0

Figure S7

Figure S7. Efficacy of antibiotic treatment and combined T cell depletion strategy (pertains to Fig.7)

A. BCG in LNs is cleared by antibiotics. One of two independent experiments. Each dot represents an individual mouse, n=5 mice per group. Paired t-test. The limit of detection = 5 CFU.

B. Frequency of total $CD90^+$ T cells (right), CD4 (middle) or CD8 (left) T cells in peripheral blood as a percentage of total live cells. Groups are untreated CC042 mice (No Tx), unvaccinated CC042 mice treated with anti-CD4 + anti-CD8α mAbs and antibiotics (Abs/Abx), BCG-vaccinated CC042 mice (BCG) or BCG-vaccinated CC042 mice treated with anti-CD4 + anti-CD8α mAbs and antibiotics (BCG/Abs/Abx).

C. Frequency of total $CD90^+$ T cells (right), CD4 (middle) or CD8 (left) T cells in peripheral blood as a percentage of total live cells. Groups are unvaccinated B6 mice (B6), unvaccinated CC042 mice (CC042), BCG-vaccinated CC042 mice (BCG) or BCG-vaccinated CC042 mice treated with anti-CD4 + anti-CD8α mAbs (CC042 BCG+T Dep).

B, C. Data is representative of 2 experiments with similar results. Each point is an individual subject, n=4-9. Bars, mean. One-way ANOVA with Šídák's multiple comparisons test. Box plots indicate median (middle line), 25th, 75th percentile (box) and minimum and maximum (whiskers).

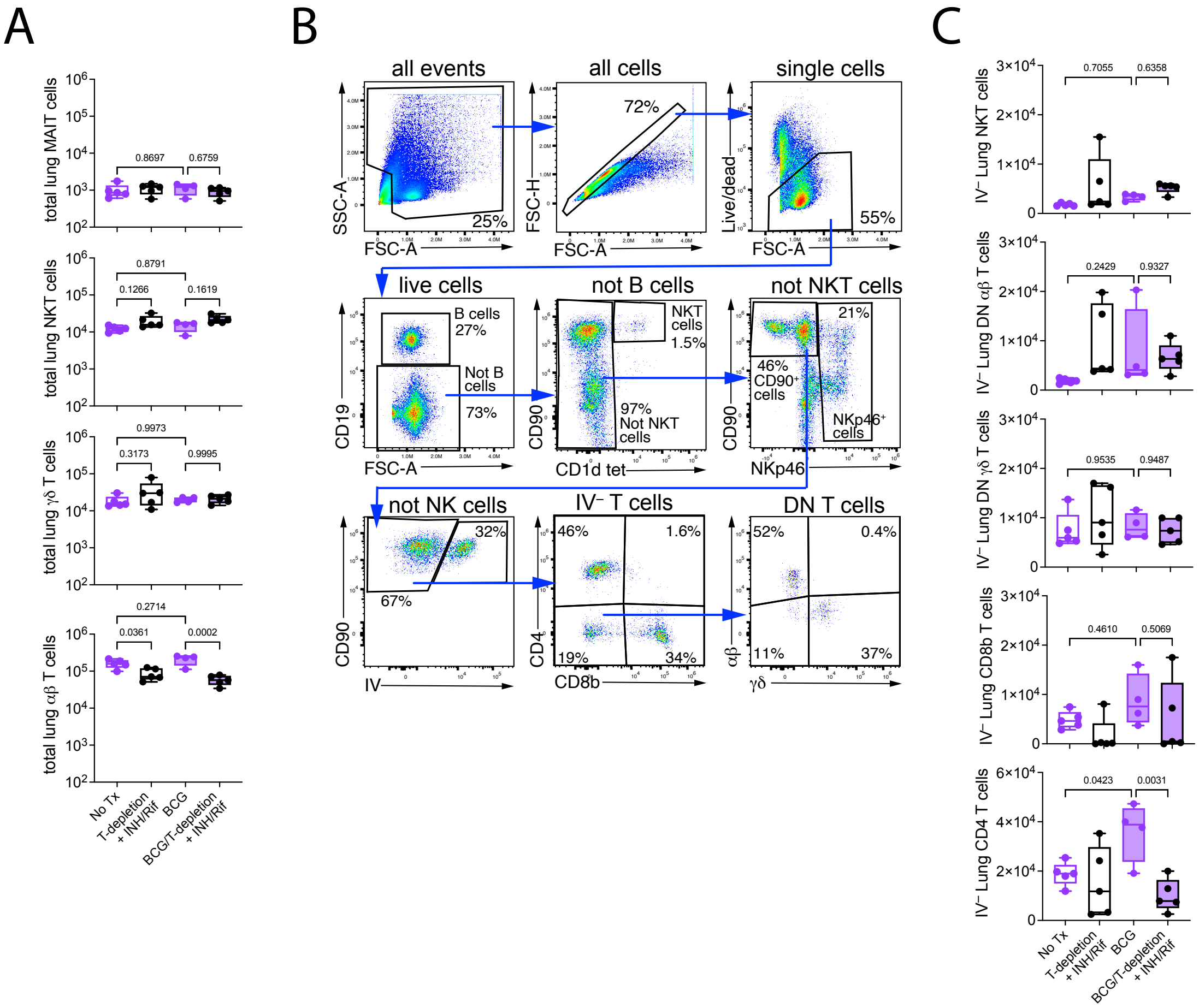
A
total lung MAIT cells
0.8697
0.6759
total lung NKT cells
0.8791
0.1266
0.1619
total lung γδ T cells
0.9973
0.3173
0.9995
total lung αβ T cells
0.2714
0.0361
0.0002
No Tx
T-depletion + INH/Rif
BCG
BCG/T-depletion + INH/Rif
B
all events
25%
SSC-A
FSC-A
all cells
72%
FSC-H
FSC-A
single cells
55%
Live/dead
FSC-A
live cells
B cells 27%
Not B cells 73%
CD19
FSC-A
not B cells
NKT cells 1.5%
97% Not NKT cells
CD90
CD1d tet
not NKT cells
21%
46% CD90+ cells
NKp46+ cells
CD90
NKp46
not NK cells
32%
67%
CD90
IV
IV− T cells
46%
1.6%
19%
34%
CD4
CD8b
DN T cells
52%
0.4%
11%
37%
αβ
γδ
C
IV− Lung NKT cells
0.7055
0.6358
IV− Lung DN αβ T cells
0.2429
0.9327
IV− Lung DN γδ T cells
0.9535
0.9487
IV− Lung CD8b T cells
0.4610
0.5069
IV− Lung CD4 T cells
0.0423
0.0031
No Tx
T-depletion + INH/Rif
BCG
BCG/T-depletion + INH/Rif

Figure S8. T cells in the lung parenchyma after Mtb infection (pertains to Fig.7)

A. Different types of T cells were enumerated in the lungs of mice treated as shown in Fig.7D-E, 4 weeks after Mtb challenge. MAIT and NKT cells identified using tetramers.

B. Gating strategy to measure different T cell classes and their location in the lung. Intravascular cells were identified by injecting fluorochrome labelled antibodies intravenously, 3 minutes prior to euthanasia.

C. Number of lung parenchyma (i.e., IV^{-}) T cell subsets in unvaccinated and BCG vaccinated CC042 mice after treatment (see Fig.7D), measured four weeks after Mtb infection.

Representative result from one of two independent experiments using untreated CC042 mice (No Tx), unvaccinated CC042 mice treated with anti-CD4 + anti-CD8α mAbs and antibiotics (T-depletion + INH/Rif), BCG-vaccinated CC042 mice (BCG) or BCG-vaccinated CC042 mice treated with anti-CD4 + anti-CD8α mAbs and antibiotics (T-depletion + INH/Rif). Each dot represents an individual mouse, n=4-5 mice per group. One-way ANOVA with Šídák's multiple comparisons test. Box plots indicate median (middle line), 25th, 75th percentile (box) and minimum and maximum (whiskers).

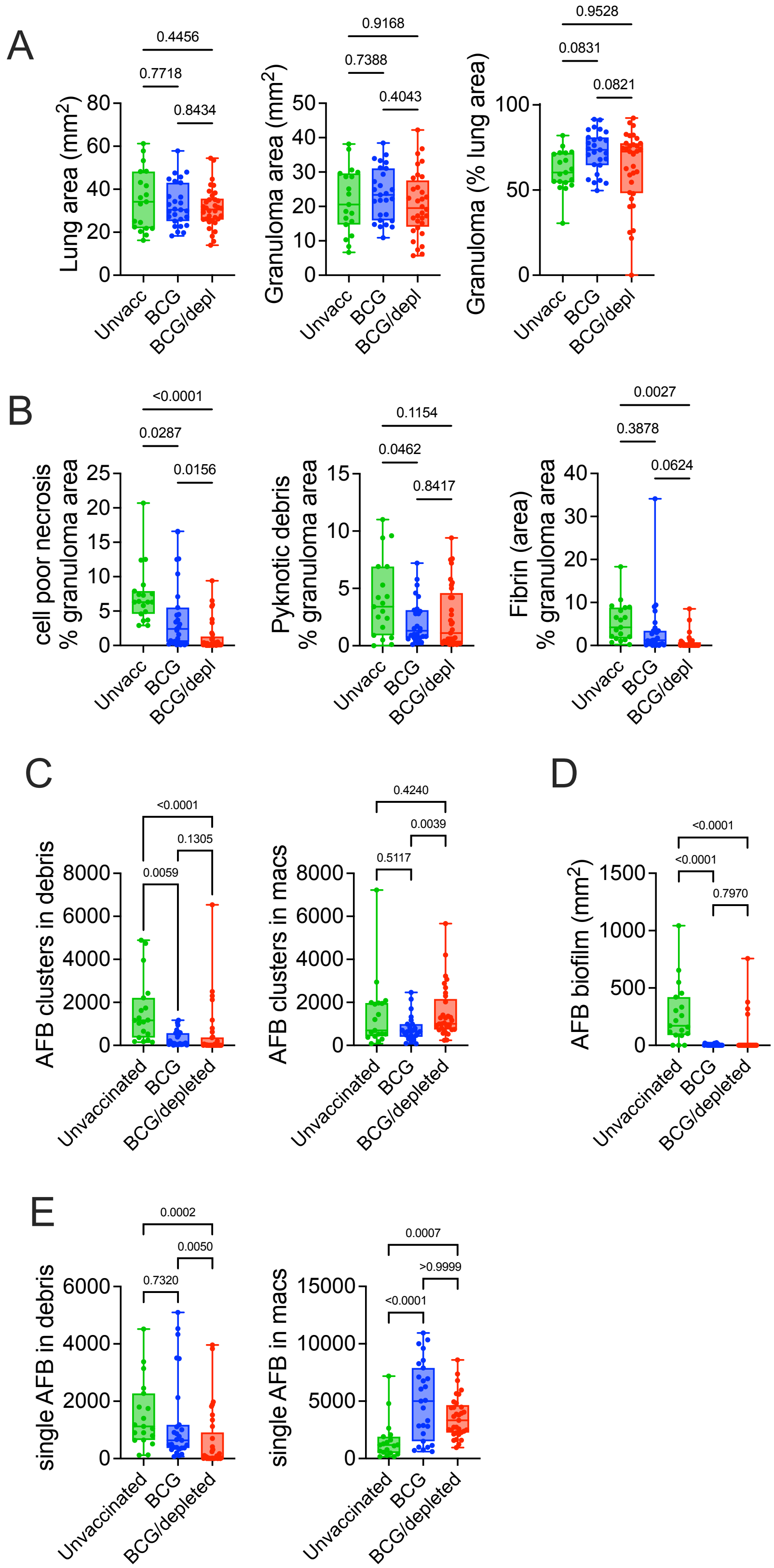
A
Lung area (mm^2)
0.4456
0.7718
0.8434
Granuloma area (mm^2)
0.9168
0.7388
0.4043
Granuloma (% lung area)
0.9528
0.0831
0.0821
Unvacc
BCG
BCG/depl
B
cell poor necrosis % granuloma area
<0.0001
0.0287
0.0156
Pyknotic debris % granuloma area
0.1154
0.0462
0.8417
Fibrin (area) % granuloma area
0.0027
0.3878
0.0624
C
AFB clusters in debris
<0.0001
0.1305
0.0059
AFB clusters in macs
0.4240
0.0039
0.5117
D
AFB biofilm (mm^2)
<0.0001
<0.0001
0.7970
E
single AFB in debris
0.0002
0.0050
0.7320
single AFB in macs
0.0007
>0.9999
<0.0001
Unvaccinated
BCG
BCG/depleted

Figure S9. Histological analysis (pertains to Fig.8)

A-D. Automated image analysis of histopathological tissue**.** Three to five lung lobes were analyzed per mouse for a total of 19 (unvaccinated), 27 (BCG), and 34 (BCG+T depletion). **A**. The total area of each scanned lung (left), the total area occupied by lesional tissue (i.e., granulomas, center), and the percentage of the lung occupied by granulomas (right). **B**. Analysis of parameters related to tissue death and destruction including the percentage of lung occupied by cell poor necrosis (left), the percentage of pyknotic debris (middle), and the amount of fibrin (right). **C**. Measurement of parameters relating to the bacterial burden including, from left to right, the number of AFB clusters in debris or in macrophages. the number of single Mtb in debris or macrophages. **D**. The absolute area of AFB biofilm. **E**. The number of single Mtb in debris (left) or macrophages (right).

A-E. Comparisons between different groups were analyzed using a non-parametric one-way Anova (Kruskal-Wallis). Box plots indicate median (middle line), 25th, 75th percentile (box) and minimum and maximum (whiskers).

Supplemental Table 1. Antibodies

Specificity	Fluor	Clone	Dilution	Vendor	Catalog #
B220	BV570	RA3-6B2	1:200	Biolegend	103237
CD103	BB700	2E7	1:200	BD Biosciences	748240
CD11a	APC	121/7	1:200	Biolegend	153110
CD11a	BV711	M17/4	1:200	BD Biosciences	740676
CD127	PE-Dazzle594	A7R34	1:200	Biolegend	135032
CD135	PE	A2F10	1:200	Biolegend	135305
CD150	PerCP-Cy5.5	TC15-12F12.2	1:200	Biolegend	115921
CD19	PE-Cy5	6D5	1:200	Biolegend	115510
CD19	APC-Cy7	6D5	1:200	Biolegend	115578
CD19	AF647	6D5	1.5ug per mouse	Biolegend	115522
CD21/CD35	AF594	7E9	1:200	Biolegend	123426
CD23	APC/Cy7	B3B4	1:200	Biolegend	101629
CD3	Percp/Cy5.5	17A2	1:200	Biolegend	1002018
CD3	PE	145-2C11	1:200	Biolegend	100307
CD3	FITC	145-2C11	1:200	Biolegend	100305
CD3	Percp/Cy5.5	17A2	1:200	Biolegend	1002018
CD34	FITC	RAM34	1:200	BD Biosciences	560238
CD4	AF700	GK1.5	1:200	Biolegend	100430
CD4	BV421	RM4-5	1:200	Biolegend	100543
CD4	PE	GK1.5	1:200	Biolegend	100408
CD4	APC	GK1.5	1:200	Biolegend	100412
CD4	PerCPCy5.5	GK1.5	1:200	Biolegend	100434
CD4	AF700	RM4-5	1:200	BD Biosciences	557956
CD44	PerCPcy5.5	IM7	1:200	Biolegend	103032
CD44	PE-Cy7	IM7	1:200	Biolegend	103036
CD44	APC	IM7	1:200	Biolegend	103012
CD44	PE	IM7	1:200	Biolegend	103007
CD48	Pacific Blue	HM48-1	1:200	Biolegend	103417
CD49b	PE/Cy7	DX5	1:200	Biolegend	108922
CD62L	BV570	MEL14	1:200	Biolegend	104433
CD62L	FITC	MEL-14	1:200	Biolegend	104406
CD62L	BV570	MEL14	1:200	Biolegend	104433
CD69	PE	H1.2F3	1:200	Biolegend	104508
CD69	PE/Cy7	H1.2F3	1:200	Biolegend	104512
CD8a	APC/Fire 750	53-6.7	1:200	Biolegend	100766
CD8a	BV421	53-6.7	1:200	Biolegend	100738
CD8a	BV510	53-6.7	1:200	Biolegend	100752
CD8b	APC-Fire 750	YTS156.7.7	1:200	Biolegend	126626
CD90.2	Pacific Blue	30-H12	1:200	Biolegend	105324
CD90.2	APC	53.2.1	1:200	Biolegend	140312
CD90.2	Pacific Blue	30-H12	1:200	Biolegend	105324
CD90.2	BV510	30-H12	1:200	Biolegend	140319
CD90.2	AF647	30-H12	1.5ug per mouse	Biolegend	105318

CD90.2	FITC	30-H12	1.5ug per mouse	Invitrogen/Fisher	11-0903-82
CD93	FITC	AA4.1	1:200	Biolegend	136508
c-kit	APC	2B8	1:200	Biolegend	105811
IgD	BV711	11-26c.2a	1:200	Biolegend	405731
IgM	PE	RMM-1	1:200	Biolegend	406507
lineage	AF700	N/A	1:200	Biolegend	79923
NK1.1	PerCP/Cy5.5	PK136	1:200	Biolegend	108702
NKp46	PE/Cy7	29A1.4	1:200	Biolegend	137618
NKp46	AF647	29A1.4	1.5ug per mouse	Biolegend	137628
Sca-1	PE-Cy7	D7	1:200	Biolegend	108113
TCRb	FITC	H57-597	1:200	Biolegend	109206
TCRb	APC	H57-597	1:200	Biolegend	109212
TCRgd	BV650	GL3	1:200	Biolegend	746962
TCRgd	PE/Cy7	GL3	1:200	Biolegend	118124
